# Unusual magnetotransport in twisted bilayer graphene from strain-induced open Fermi surfaces

**DOI:** 10.1073/pnas.2307151120

**Published:** 2023-08-14

**Authors:** Xiaoyu Wang, Joe Finney, Aaron L. Sharpe, Linsey K. Rodenbach, Connie L. Hsueh, Kenji Watanabe, Takashi Taniguchi, M. A. Kastner, Oskar Vafek, David Goldhaber-Gordon

**Affiliations:** ^a^National High Magnetic Field Laboratory, Tallahassee, FL 32310; ^b^Department of Physics, Stanford University, Stanford, CA 94305; ^c^Stanford Institute for Materials and Energy Sciences, SLAC National Accelerator Laboratory, Menlo Park, CA 94025; ^d^Materials Physics Department, Sandia National Laboratories, Livermore, CA 94550; ^e^Department of Applied Physics, Stanford University, Stanford, CA 94305; ^f^Research Center for Functional Materials, National Institute for Materials Science, Tsukuba 305-0044, Japan; ^g^International Center for Materials Nanoarchitectonics, National Institute for Materials Science, Tsukuba 305-0044, Japan; ^h^Department of Physics, Massachusetts Institute of Technology, Cambridge, MA 02139; ^i^Department of Physics, Florida State University, Tallahassee, FL 32306

**Keywords:** moiré materials, heterostrain, magnetotransport, bilayer graphene

## Abstract

Anisotropic hopping in a toy Hofstadter model was recently invoked to explain a rich and surprising Landau spectrum measured in twisted bilayer graphene away from the magic angle. Suspecting that such anisotropy could arise from unintended uniaxial strain, we extend the Bistritzer–MacDonald model to include uniaxial heterostrain and present a detailed analysis of its impact on band structure and magnetotransport. We find that such strain strongly influences band structure, shifting the three otherwise-degenerate van Hove points to different energies. Coupled to a Boltzmann magnetotransport calculation, this reproduces previously unexplained nonsaturating $B^{2}$ magnetoresistance over broad ranges of density near filling ν=±2 and predicts subtler features that had not been noticed in the experimental data. In contrast to these distinctive signatures in longitudinal resistivity, the Hall coefficient is barely influenced by strain, to the extent that it still shows a single sign change on each side of the charge neutrality point—surprisingly, this sign change no longer occurs at a van Hove point. The theory also predicts a marked rotation of the electrical transport principal axes as a function of filling even for fixed strain and for rigid bands. More careful examination of interaction-induced nematic order versus strain effects in twisted bilayer graphene could thus be in order.

## Significance

Because of its rich array of correlated phases, twisted bilayer graphene (TBG) near the magic angle has captivated the condensed matter physics world. The large moiré length scale not only promotes interaction-related effects but also allows for extrinsic factors such as strain to play a major role. In a previous work, we presented measurements of a TBG device with several unusual behaviors in magnetotransport and conjectured that uniaxial strain could explain our measurements. Here, we model magnetotransport in TBG by incorporating uniaxial heterostrain into the Bistritzer–MacDonald Hamiltonian. The theory not only reproduces the unusual phenomena from the previous work but also predicts additional features unnoticed before. Our work therefore demonstrates the crucial role of heterostrain in TBG devices.

The discovery of superconductivity and correlated insulating states in magic-angle twisted bilayer graphene (TBG) (1, 2) placed the material at the forefront of condensed matter physics research (3–18). The moiré superlattice potential of TBG, resulting from a small relative twist angle θ between the graphene layers, can induce nearly flat, topologically nontrivial, isolated bands, consisting of electronic states near the Dirac points of each monolayer of graphene (19). As a result, TBG is an exceptional platform for studying the interplay of electron correlations and band topology (20–38).

Strain—especially differing lattice distortions in the two layers, termed heterostrain—is believed to play an important role in the phase diagram of TBG (37–41). Scanning probe measurements typically find uniaxial heterostrain in the range of 0.1 to 0.7% (3, 9, 11, 42) in samples fabricated with the tear-and-stack method (43, 44). For heterostrain, as opposed to strain applied equally to both layers, the linear distortion of the moiré unit cell is amplified by a factor of ∼1/θ relative to the linear distortion of the microscopic atomic lattice. For example, 0.2% uniaxial heterostrain causes an ∼8% change in the largest linear dimension of the moiré unit cell for a twist angle of $1.38^{{}^{\circ}}$. The moiré unit cell area changes by much less, only ∼0.1%, but because we infer twist angle from moiré unit cell area in transport, this dependence of area on strain still leads to underestimates of the uncertainty in twist angles presented in the transport literature, as noted in ref. 3.

In a recent report by some of the authors [Finney et al. (45)], a TBG sample with a moiré unit cell area of 90 nm${}^{2}$ (corresponding to $\theta =1.38^{{}^{\circ}}$) displayed several unusual phenomena in magnetotransport. As anticipated for a twist well above the magic angle, the sample did not exhibit the strong interaction-driven effects typically observed in near-magic-angle devices. Surprisingly, though, over a broad filling range near half-filling the longitudinal magnetoresistivity (MR) exhibited a $B^{2}$ increase up to ∼100-fold at ≈5 T, after which quantum oscillations set in. The authors found that a toy Hofstadter model with anisotropy showed multiple features similar to those in the data, and they conjectured that uniaxial strain might cause such anisotropy.

In this work, we present a systematic theoretical study of the impact of uniaxial heterostrain on the narrow-band dispersion of TBG above the magic angle, analyze its consequences for weak field magnetotransport, and compare it with experimental data from ref. 45. We base our theory on the Bistritzer–MacDonald (BM) continuum model (19), incorporating heterostrain in the form of a deformation potential, a pseudomagnetic field (46, 47), and a distortion of the moiré pattern.

Our key theoretical result is that heterostrain lifts the energetic degeneracy of the two Dirac points as well as that of the three van Hove points of a given band. The splitting of the two Dirac points leads to a semimetallic state with small Fermi pockets near the charge neutrality point (CNP). More interestingly, the splitting of the van Hove points leads to open Fermi surfaces (FSs) in the filling range bounded by two of the van Hove points. In the weak field semiclassical regime governed by the Boltzmann equation, the open FSs generally lead to a nonsaturating $B^{2}$ MR (48), accounting for this previously unexplained feature in the experimental data of ref. 45.

This theory makes a number of falsifiable predictions. 1) If the direction of current flow in the lithographically patterned Hall bar is not aligned with the 1D-like principal axis of transport in the open FS regime, longitudinal magnetoresistance should mix substantially into the measured resistance at Hall contacts. 2) A subtle cusp should appear in resistivity as filling crosses the lowest-energy van Hove point. 3) A Lifshitz transition from two FS pockets to one should also coincide with crossing this lower van Hove point. We reanalyze experimental data from ref. 45 and find that these predictions are verified. The strained BM model studied here has electron–hole symmetry. We leave the discussion of electron–hole asymmetry in the experimental data to future works.

The theory also has implications that are not so far directly probed by the experiment, but are striking. First, on each side of the CNP, the divergence and sign change in the Hall number near half-filling of a 4-fold degenerate band does not coincide with any of the van Hove points but instead occurs within the filling range where FSs are open. Second, the transport principal axis continuously rotates by up to $90^{{}^{\circ}}$ as density is tuned from the CNP to the open FS regime. Such rotation of the transport axes has been presented as evidence for interaction-induced nematic order (14), but here, we find that it can arise purely due to strain-induced band structure effects.

This work clearly demonstrates that the effects of even miniscule amounts of heterostrain in TBG cannot be neglected. Dramatic and unexpected phenomena occur in strained TBG even in the single-particle regime, without the strong correlation effects that arise near the magic angle. Given the amplifying effect of a small heterostrain on the moiré length scale, it is tantalizing to consider strain engineering of such devices to achieve effects that would be impossible in regular solids due to structural instabilities.

The paper is organized as follows: In Section I, we present the theoretical calculation of the changes in band structure resulting from uniaxial strain. In Section II, we use the Boltzmann transport equation to calculate the electron transport properties in magnetic fields resulting from the strain-induced alterations in the band structure. Section III is a comparison of our predictions with experiment, and it is sufficiently self-contained that a reader who does not need all the theoretical details can jump directly there. In Section IV, we summarize our results.

## 1. Geometric and Energetic Effects of Uniaxial Heterostrain on TBG

In the limit of small deformations, both uniaxial heterostrain and a small twist angle are captured via a coordinate transformation: $r_{l}^{\prime }=r+u_{l}\left(r\right)$, where l=t,b labels the *Top* (*Bottom*) graphene layers, and $u_{l}\left(r\right)\approx E_{l}r$ is the local deformation field. The symmetric and antisymmetric part of the 2×2 tensor $E_{l}$ describes strain and rotation, respectively. For twist angle (θ) and a uniaxial heterostrain of strength (ϵ) and direction (φ), we parameterize $E_{t}=-E_{b}\equiv E/2$, where E≡T(θ)+S(ϵ,φ), and given by:

[1] $$\begin{array}{c} T\left(\theta \right)=\left(\begin{array}{cc} 0 & -\theta \\ \theta & 0 \end{array}\right),\;S\left(\epsilon ,\varphi \right)=R_{\varphi }^{T}\left(\begin{array}{cc} -\epsilon & 0\\ 0 & \nu \epsilon \end{array}\right)R_{\varphi }. \end{array}$$

**Fig. 1.**
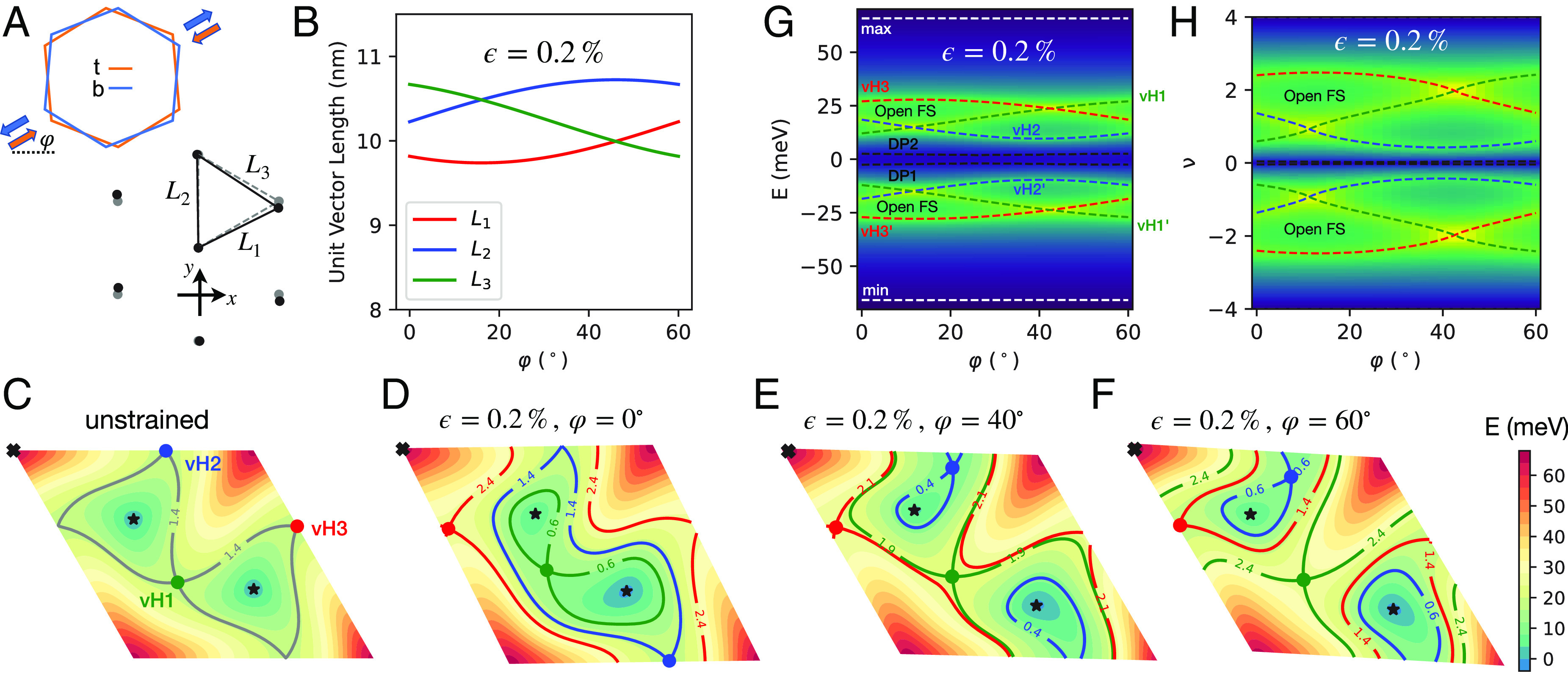
(*A*) Schematics of applying a uniaxial heterostrain on the pair of microscopic unit cells of monolayer graphene making up TBG. (*Upper* sketch) Orange (blue) color corresponds to the *Top* (*Bottom*) layer. The uniaxial strain of strength +(−)ϵ/2 and direction φ on the *Top* (*Bottom*) layer are represented as colored arrows. (*Lower* sketch) Deformation of the moiré superlattice for twist angle $1.38^{{}^{\circ}}$ due to a uniaxial heterostrain of ϵ=0.2% and $\varphi =0^{{}^{\circ}}$. Unstrained (gray, dashed) and strained (black, solid) triangular lattice sites of AA stacking regions of the moiré superlattice are depicted. (*B*) Dependence of the three moiré triangular bond lengths on φ for a fixed strength. (*C*–*F*) Energy maps of the *Upper* band of the BM Hamiltonian in valley K, plotted in the moiré Brillouin zone specified by $k=k_{1}g_{1}+k_{2}g_{2}$, where $k_{1,2}\in \left[0,1\right)$. There are six special points of the band structure, i.e., two Dirac points (black stars), three van Hove points (colored dots), and one band maximum (black cross). The contour lines intersecting the van Hove points are plotted and labeled by their respective filling fractions. In the unstrained case (*C*), the two Dirac points and three van Hove points are respectively at equal energies. The energy degeneracies are lifted in the presence of uniaxial heterostrain, as illustrated in (*D*–*F*). This leads to semimetallic behavior at the CNP, and a φ-dependent filling range near ν=2 with open FSs. (*G* and *H*) φ-dependence of the energies and filling fractions of the band structure special points for a fixed heterostrain strength. The background colormap is the calculated density of states, with a broadening of δ=1meV. Green (blue) color represents high (low) density of states. The energetic minimum and maximum of the narrow bands are shown with horizontal dashed gray lines.

Here, $R_{\varphi }$ is the two-dimensional rotation matrix, and ν≈0.16 is the Poisson ratio (3). Physically, ϵ>0 corresponds to compressing the *Top* layer while stretching the *Bottom* layer along the direction determined by φ, as illustrated in Fig. 1*A*. A relative deformation E between the graphene bilayers generates a moiré superlattice, with moiré reciprocal lattice vectors $g_{i=1,2}=E^{T}G_{i=1,2}$, where $G_{i}$ are reciprocal lattice vectors of the undeformed monolayer graphene. The moiré lattice vectors $L_{i=1,2}$ are uniquely defined through the relation $L_{i}\cdot g_{j}=2\pi \delta_{\mathrm{ij}}$. It is important to note that only relative deformations generate the moiré superlattice. Deformations that act homogeneously on the two layers have little effect on the narrow band physics, and we accordingly neglect them in this work^*^.

Under rotation $R_{\varphi }$, the strain tensor transforms as a headless vector that remains invariant under $\varphi \to \varphi +180^{{}^{\circ}}$. Combined with the $C_{3z}$ symmetry of the undeformed graphene lattice, the strained electronic dispersion within a given graphene valley simply rotates $60^{{}^{\circ}}$ under $\varphi \to \varphi +60^{{}^{\circ}}$. We therefore only report results for $\varphi \in [0^{{}^{\circ}},60^{{}^{\circ}})$. For concreteness, we define the microscopic unit cell vectors $a_{i=1,2}$ of an undeformed graphene lattice as $a_{1}=a\left(\frac{1}{2},-\frac{\sqrt{3}}{2}\right),\;a_{2}=a\left(1,0\right)$, where a≈2.46 Å is the lattice constant. The positions of the sublattice A, B within a unit cell are chosen as ${\vec{\tau }}_{A}=\left(0,0\right)$ and ${\vec{\tau }}_{B}=\frac{a}{\sqrt{3}}\left(0,1\right)$. The reciprocal lattice vectors are $G_{1}=\frac{4\pi }{\sqrt{3}a}\left(0,-1\right)$ and $G_{2}=\frac{4\pi }{\sqrt{3}a}\left(\frac{\sqrt{3}}{2},\frac{1}{2}\right)$. Different conventions lead to different definitions of the Dirac Hamiltonian (see for instance ref. 40), but the physics is consistent.

Fig. 1 *A* and *B* illustrates the geometric effects of heterostrain for twist angle $\theta =1.38^{{}^{\circ}}$. At ϵ=0, AA-stacked regions of the moiré form an equilateral triangular superlattice. Introducing uniaxial heterostrain changes the spacings between neighboring AA-stacked regions ($L_{i=1,2,3}$). For ϵ=0.2%, typical in these systems (3, 9, 11), the variation in spacings can be as large as ϵ/θ≈8%. Such dramatic amplification of the microscopic strain makes moiré materials uniquely suited to strain engineering—conventional materials become structurally unstable at distortions only 10% as large as those achieved in the moiré superlattice. Note that the effect of uniaxial heterostrain on the moiré unit cell area is small at $\nu^{2}\epsilon^{2}/\theta^{2}$, as some spacings become larger while others become smaller (*SI Appendix*, section I).

We proceed to discuss the energetic effects in the context of the continuum BM model (19). We work in the limit where both $E_{l}$ and the wavevector k in the moiré Brillouin zone are small and consider only the leading order terms in both. This would mean, for instance, that terms such as Ek are omitted as higher-order terms. This treatment is generally justified away from the magic angle because higher-order terms can play an important role only close to the magic angle where the bandwidth becomes comparable to these terms (49, 50). We explicitly checked that at $\theta \approx 1.38^{{}^{\circ}}$, the effects of such higher-order terms are indeed negligibly small. To leading order, the strained BM Hamiltonian for a given valley is given by

[2] $$\begin{array}{c} H_{\eta }=\left(\sum_{l=t,b}H_{\eta ,l}^{\mathrm{intra}}\right)+H_{\eta }^{\mathrm{inter}}, \end{array}$$

where η=±1 labels $K\;(K^{\prime })$ valleys of monolayer graphene. The interlayer Hamiltonian is given by

[3] $$\begin{array}{c} H_{\eta }^{\mathrm{inter}}\approx \int d^{2}r\psi_{\eta ,t}^{†}\left(r\right)\left(\sum_{j=1,2,3}T_{\eta ,j}e^{-i\eta q_{j}\cdot r}\right)\psi_{\eta ,b}\left(r\right)+h.c., \end{array}$$

where $\psi_{\eta ,l}\left(r\right)\equiv {\left(\psi_{\eta ,l,A}\left(r\right),\psi_{\eta ,l,B}\left(r\right)\right)}^{T}$ is a spinor in the sublattice basis for a given valley and layer. We have suppressed the spin index for simplicity. $q_{j=1,2,3}$ are the three nearest neighbor bonds of the reciprocal honeycomb lattice, and

[4] $$\begin{array}{c} T_{\eta ,j}=w_{0}\sigma_{0}+w_{1}\left(\cos\frac{2\pi (j-1)}{3}\sigma_{x}+\eta \sin\frac{2\pi (j-1)}{3}\sigma_{y}\right). \end{array}$$

$\left(\sigma_{0},\sigma_{x},\sigma_{y}\right)$ are Pauli matrices acting on sublattice degrees of freedom.

The intralayer Hamiltonian is given by:

[5] $$\begin{array}{c} \begin{array}{cc} & H_{\eta ,l}^{\mathrm{intra}}=\alpha \int d^{2}r\psi_{\eta ,l}^{†}\left(r\right)\left(\;\text{tr}\left[E_{l}\right]\sigma_{0}\right)\psi_{\eta ,l}\left(r\right)\\ & -\frac{\hbar v_{F}}{a}\int d^{2}r\psi_{\eta ,l}^{†}\left(r\right)\left[\left(-i\nabla -A_{\eta ,l}\right)\cdot \left(\eta \sigma_{x},\sigma_{y}\right)\right]\psi_{\eta ,l}\left(r\right). \end{array} \end{array}$$

Here, the first term is the deformation potential that couples to the electron density. Its value is not precisely known in the literature, with numbers ranging from −4.1eV to 30eV depending on the methodology (51–54). We use α=−4.1eV in this work based on first principles calculations (54), although for heterostrain ϵ≈0.2% varying the deformation potential over the range proposed in the literature leads to only minor quantitative differences in band dispersions. $A_{\eta ,l}$ is the pseudovector potential that comes from changes in the intersublattice hopping due to deformations and changes sign between graphene valleys. It is given as refs. 46 and 47: $A_{\eta ,l}=\frac{\sqrt{3}\beta }{2a}\eta \left(\epsilon_{l,xx}-\epsilon_{l,yy},-2\epsilon_{l,xy}\right)$, where we choose β≈3.14 from refs. 3 and 40. We further fix $\hbar v_{F}/a=2.68\mathrm{eV}$ based on Fermi velocity in monolayer graphene $v_{F}\approx 10^{6}m/s$ (55), $w_{0}=88\mathrm{meV}$, and $w_{1}=110\mathrm{meV}$ (19) in our calculations and also set ħ=1 in the remainder of the paper.

To leading order approximation, the strained BM Hamiltonian in a given valley (Eq. **2**) has particle–hole symmetry under $P\psi_{l}\left(r\right)=\sum_{l^{\prime }}i{\left(\mu_{y}\right)}_{ll^{\prime }}\psi_{l^{\prime }}\left(-r\right)$ (56), where $\mu_{y}$ is a Pauli matrix acting on the layer degrees of freedom. This means that for every single-electron state at energy E and wavevector k, there is a state at energy −E and wavevector −k. This particle–hole symmetry has been investigated extensively for the unstrained BM model, e.g., refs. 26 and 57, and here, it is generalized to the strained case. The source of experimentally evident particle–hole asymmetry in the off-magic-angle device (45) could be higher-order gradient terms beyond what is captured in the BM model in Eq. **2**, interaction effects (58–61), or their combination.

Fig. 1*D*–*F* displays the effects on the band structure of ϵ=0.2% heterostrain applied in select directions relative to the x-axis defined in Fig. 1*A*, as specified by $\varphi \in [0^{{}^{\circ}},60^{{}^{\circ}})$. For simplicity, we show only contour maps of the upper band from valley K in the moiré Brillouin zone specified by $k=k_{1}g_{1}+k_{2}g_{2}$, where $k_{1,2}\in \left[0,1\right)$. Heterostrain preserves $C_{2}T$ and valley U(1) (23), so the *Lower* and *Upper* bands remain connected via two Dirac points. The *Upper* band features six special points—two Dirac points (black stars), three van Hove points (colored dots), and one band maximum (black cross). The six special points of a given band are related to “critical points” in the context of the Morse theory, which states that

[6] $$\begin{array}{c} \sum_{i}{\left(-1\right)}^{\gamma_{i}}=\chi , \end{array}$$

where $\gamma_{i}$ is the index of the i-th critical point, and χ is the Euler characteristic of a manifold (62); χ vanishes for the Brillouin zone which is a torus. Although a Dirac point is strictly a point of nonanalyticity and is not directly covered by Morse theory, if we imagine adding a tiny gap term, it will become a legitimate band extremum allowing Morse theory to apply. Whereas the two band minima (Dirac points) and the band maximum have even γ, and so each contributes +1 to the sum, every conventional van Hove point (i.e., not a higher order) has an odd γ and contributes −1. The overall sum thus vanishes. Therefore, the van Hove points can only be annihilated/created by colliding with local minima/maxima. For a relatively small heterostrain as shown in Fig. 1, the number of special points per band is the same as at ϵ=0. However, for larger heterostrain (e.g., ϵ=0.5%, see *SI Appendix*, Fig. S1), more striking behavior of the special points can occur, such as a change in their total number via aforementioned collisions and the appearance of tilted type II Dirac cones (63, 64).

A key finding of the present work is that the respective energy degeneracies of the two Dirac points and the three van Hove points are lifted by uniaxial heterostrain, by amounts depending sensitively on φ. In the absence of strain, Fig. 1*C*, the three van Hove points are at equal energy, and separate closed contours of constant energy centered around the Dirac points from closed contours centered around the band maximum. As illustrated in Fig. 1*D*–*F*, uniaxial heterostrain splits the energy degeneracy of the two Dirac points, leading to a semimetallic state with small Fermi pockets near the CNP (40). The three van Hove points also split in energy. The two outermost van Hove points (i.e., closer to the band maximum) bound a filling range of open FSs near ν=2, whereas the innermost van Hove point moves closer to one of the Dirac points. If we continue increasing ϵ, a collision of the critical points occurs, the innermost van Hove disappears, the two Dirac points become type II tilted, and a new ordinary minimum is created. Note that a small mass added to type II tilted Dirac points will not introduce band extrema, and as a consequence, type II tilted Dirac points are not critical points of Morse theory. Therefore, after the collision, Eq. **6** still holds.

Interestingly, the elongation of the FSs shows a strong filling dependence. Close to the CNP, the bigger Fermi pocket that encloses a Dirac point is stretched along a direction perpendicular to that of the open FSs; see Figs. 1*D*–*F*. As explained later, this leads to a dramatic rotation of the principal transport axis when the filling is tuned from the CNP to the open FS range.

The dependence of the energy and filling of the band structure special points on φ at a fixed ϵ is shown in Fig. 1 *G* and *H*. Of notable interest is the sensitivity of the filling range with open FSs to φ. This filling range must in fact vanish at some φ between $0^{{}^{\circ}}$ and $60^{{}^{\circ}}$, when the energies of the two outermost van Hove points cross. As seen in Fig. 1*D*–*F*, this also alters the elongation of the open FSs.

## 2. Boltzmann Equation and Magnetoresistivity in TBG

Having understood the heterostrain effects on the bandstructure, we proceed to discuss the implications for magnetotransport. We begin by considering the general structure of the two-dimensional resistivity tensor ρ subject to heterostrain. The resistivity tensor is defined via

[7] $$\begin{array}{c} \left(\begin{array}{c} E_{x}\\ E_{y} \end{array}\right)=\left(\begin{array}{cc} \rho_{\mathrm{xx}} & \rho_{\mathrm{xy}}\\ \rho_{\mathrm{yx}} & \rho_{\mathrm{yy}} \end{array}\right)\left(\begin{array}{c} j_{x}\\ j_{y} \end{array}\right), \end{array}$$

where $E={\left(E_{x},E_{y}\right)}^{T}$ and $j={\left(j_{x},j_{y}\right)}^{T}$ are electric field and current vectors, respectively. Under rotation by δθ, the resistivity tensor transforms as:

[8] $$\begin{array}{c} \rho^{\prime }=R_{\delta \theta }^{T}\rho R_{\delta \theta },\;R_{\delta \theta }=\left(\begin{array}{cc} \cos\delta \theta & -\sin\delta \theta \\ \sin\delta \theta & \cos\delta \theta \end{array}\right). \end{array}$$

If the underlying system has a point group symmetry higher than $C_{2z}$ (e.g., $C_{3z},C_{6z}$), then $\rho =\rho_{0}I-i\rho_{H}\tau_{y}$ is the most general form of ρ invariant under such rotations. Here, $\tau_{y}$ is the Pauli matrix acting in the two-dimensional coordinate basis, $\rho_{0}\left(-B\right)$ = $\rho_{0}\left(B\right)$ is the longitudinal resistivity, and $\rho_{H}\left(-B\right)$ = $-\rho_{H}\left(B\right)$ is the Hall resistivity. The even/odd parity under time reversal is guaranteed by the Onsager reciprocal relations.

Since heterostrain breaks the point group symmetry down to $C_{2z}$, we generally expect $\rho_{\mathrm{xx}}\neq \rho_{\mathrm{yy}},\;\rho_{\mathrm{xy}}\neq -\rho_{\mathrm{yx}}$. Nevertheless, it is always possible to define transport principal axes after a suitable rotation δθ of the coordinate system, such that

[9] $$\begin{array}{c} \rho_{\;\text{principal}}=\frac{1}{2}\left(\rho_{1}+\rho_{2}\right)I+\frac{1}{2}\left(\rho_{1}-\rho_{2}\right)\tau_{z}+\rho_{H}i\tau_{y}. \end{array}$$

Here, $\rho_{1,2}$ are longitudinal resistivities along the principal transport directions ${\hat{e}}_{1,2}$, respectively. The rotation angle δθ is determined up to $180^{{}^{\circ}}$ by requiring $\rho_{1}<\rho_{2}$.

Below, we first derive the MR tensor using a Boltzmann approach for a general noninteracting electronic system within the relaxation time approximation. Since there is currently limited understanding of the scattering mechanisms determining electrical transport in TBG, here, we follow ref. 65 and use the relaxation time approximation. We then present the results for heterostrained TBG, showing that in the open FS region, the low-resistivity principal axis (${\hat{e}}_{1}$) is nearly perfectly aligned with the shortest moiré bond direction. However, there is a dramatic rotation of the principal axis as the filling moves toward the CNP. We further show that the open FSs lead to a $B^{2}$ nonsaturating MR along ${\hat{e}}_{2}$ and a saturating resistivity along ${\hat{e}}_{1}$. For random orientation ($\theta_{0}$) of the principal axis to the electrical current axis in the Hall bar geometry, e.g., as in ref. 45, the longitudinal resistivity is given by: $\rho_{\mathrm{xx}}=\rho_{1}\cos^{2}\theta_{0}+\rho_{2}\sin^{2}\theta_{0}$. This is dominated by the $\rho_{2}∼B^{2}$ component. As a result, the experimental measurements should observe the nonsaturating MR component if there is a misalignment of the Hall bar orientation with respect to the principal transport axis. Such misalignment is generically to be expected and indeed must occur over most of the relevant filling range since the principal axes rotate with filling while the device geometry remains fixed.

### Boltzmann Equation and Method of Characteristics.

We begin with a brief description of the method of characteristics used to solve the Boltzmann equation perturbatively in electric field E but without a restriction on the strength of the perpendicular magnetic field $B=B\hat{z}$, as long as the semiclassical regime holds (66). Due to $C_{2z}T$ symmetry of TBG at B=0, there is no Berry curvature contribution to the semiclassical equations of motion. Then, within the relaxation time approximation, the Boltzmann equation for a given energy band becomes

[10] $$\begin{array}{c} \frac{\partial n_{k}}{\partial t}+\left(qE+qv_{k}\times B\right)\cdot \frac{\partial n_{k}}{\partial k}=-\frac{n_{k}-n_{0,k}}{\tau }, \end{array}$$

where $qE+qv_{k}\times B$ is the total force on the Bloch electrons, with $v_{k}\equiv \nabla_{k}\varepsilon_{k}$ and charge q; $n_{0,k}$ is the equilibrium Fermi–Dirac distribution and $n_{k}$ is the desired nonequilibrium distribution function.

We consider a stationary solution to the Boltzmann equation by parameterizing the distribution function as:

[11] $$\begin{array}{c} n_{k}=n_{0,k}+n_{1,k}. \end{array}$$

As a result, the Boltzmann equation for the deviation of the distribution function from equilibrium is:

[12] $$\begin{array}{c} \left(qE\cdot v_{k}\right)\frac{\partial n_{0,k}}{\partial \varepsilon_{k}}+\left(qv_{k}\times B\right)\cdot \frac{\partial n_{1,k}}{\partial k}=-\frac{n_{1,k}}{\tau }. \end{array}$$

Note that the magnetic field only couples to $n_{1}$ since $\left(qv_{k}\times B\right)\cdot \nabla_{k}n_{0,k}=\left(qv_{k}\times B\right)\cdot v_{k}\partial_{\varepsilon_{k}}n_{0,k}=0$.

To solve the above partial differential equation (PDE), we seek a family of curves covering the k-space which we parameterize as k(s) with $s\in [0,s_{0})$, such that along these curves, the PDE becomes an ordinary differential equation (ODE). If a curve k(s) satisfies

[13] $$\begin{array}{c} \frac{dk(s)}{\mathrm{ds}}=qv\left(s\right)\times B, \end{array}$$

then $n_{1,k(s)}\equiv n_{1}\left(s\right)$ satisfies

[14] $$\begin{array}{c} \left(qE\cdot v_{k}\right)\frac{\partial n_{0,k}}{\partial \varepsilon_{k}}{|}_{k=k(s)}+\frac{dn_{1}\left(s\right)}{\mathrm{ds}}=-\frac{n_{1}\left(s\right)}{\tau }. \end{array}$$

Because

[15] $$\begin{array}{c} \frac{d\varepsilon (s)}{\mathrm{ds}}=v\left(s\right)\cdot \frac{dk(s)}{\mathrm{ds}}=0, \end{array}$$

the curve k(s) must coincide with the contour of constant energy. Thus, the Boltzmann equation becomes:

[16] $$\begin{array}{c} \left[qE\cdot v\left(s\right)\right]\frac{\partial n_{0}\left(s\right)}{\partial \varepsilon (s)}+\frac{dn_{1}\left(s\right)}{\mathrm{ds}}=-\frac{n_{1}\left(s\right)}{\tau }. \end{array}$$

The ODE is readily solved with

[17] $$\begin{array}{c} n_{1}\left(s\right)=\chi_{0}e^{-s/\tau }-e^{-s/\tau }\int_{0}^{s}ds^{\prime }e^{s^{\prime }/\tau }\left[qE\cdot v\left(s^{\prime }\right)\right]\frac{\partial n_{0}\left(s^{\prime }\right)}{\partial \varepsilon (s^{\prime })}. \end{array}$$

where $\chi_{0}$ is a constant determined by the following argument. Since k(s) describes a constant energy contour in a two-dimensional Brillouin zone, it is either a closed contour or several open contours that terminate on boundaries of the Brillouin zone such that they form a closed loop on a torus. In either case, k(s) is periodic under $s\to s+s_{0}$ modulo a moiré reciprocal lattice vector, where $s_{0}$ is the periodicity. The periodicity condition $n_{1}\left(s_{0}\right)=n_{1}\left(0\right)$ leads to

[18] $$\begin{array}{c} \chi_{0}=\frac{1}{1-e^{s_{0}/\tau }}\int_{0}^{s_{0}}ds^{\prime }e^{s^{\prime }/\tau }\left(qE\cdot v\left(s^{\prime }\right)\right)\frac{\partial n_{0}\left(s^{\prime }\right)}{\partial \varepsilon (s^{\prime })}, \end{array}$$

which determines the desired $n_{1}\left(s\right)$.

In the low-temperature limit, the steady-state current from a given energy band is calculated as:

[19] $$\begin{array}{c} \begin{array}{cc} j^{\mu } & =q\int \frac{d^{2}k}{{\left(2\pi \right)}^{2}}v_{k}^{\mu }n_{1,k}=\frac{q^{2}B}{{\left(2\pi \right)}^{2}}\int d\varepsilon \int_{0}^{s_{0}}dsv^{\mu }\left(s\right)n_{1}\left(s\right)\\ & =\frac{q^{3}B}{\left(2\pi \right)}\frac{\tau }{\omega_{c}}\sum_{n=-\infty }^{\infty }\frac{v_{n}^{\mu }v_{-n}^{\nu }}{1+in\omega_{c}\tau }E^{\nu }, \end{array} \end{array}$$

where (μ,ν)=x,y, and we have defined the cyclotron frequency as:

[20] $$\begin{array}{c} \omega_{c}\equiv 2\pi /s_{0}. \end{array}$$

We have also made use of the periodicity of velocity under $s\to s+s_{0}$ to write it in terms of Fourier series, $v\left(s\right)=\sum_{n=-\infty }^{\infty }v_{n}e^{-in\omega_{c}s}$.

To show that the second line of Eq. **19** holds, note that at every k, we can define a local coordinate system $\left({\hat{e}}_{v},{\hat{e}}_{s}\right)$ such that $v\equiv v{\hat{e}}_{v}$ where v≥0, and ${\hat{e}}_{s}={\hat{e}}_{v}\times \hat{z}$. The infinitesimal wavevector can be equivalently written as:

$$dk=dk_{x}{\hat{e}}_{x}+dk_{y}{\hat{e}}_{y}=dk_{s}{\hat{e}}_{s}+dk_{v}{\hat{e}}_{v}.$$

Eq. **13** can then be written as $dk/ds=qvB{\hat{e}}_{s}$, or equivalently $dk_{s}=qvBds$. As a result,

$$\int dk_{x}dk_{y}=\int dk_{s}dk_{v}=qB\int d\varepsilon ds.$$

The conductivity tensor is therefore given by the following expression:

[21] $$\begin{array}{c} \sigma^{\mu \nu }=\frac{q^{3}B}{2\pi }\frac{\tau }{\omega_{c}}\sum_{n=-\infty }^{\infty }\frac{v_{n}^{\left(\mu \right)}v_{-n}^{\left(\nu \right)}}{1+in\omega_{c}\tau }. \end{array}$$

Eq. **21** gives the magnetoconductivity for a given FS contour. In the case of multiple FS contours and multiple bands—associated for example with spin and valley degeneracy in TBG—conductivities from different FS contours and bands add. Finally, the MR tensor is obtained by inverting the conductivity tensor, i.e., $\rho ={\left(\sum_{n,i}\sigma_{n,i}\right)}^{-1}$, where n,i are band and contour labels respectively for a given energy level.

To better understand Eq. **21**, consider an example of a parabolic dispersion with $\varepsilon_{k}=\frac{1}{2m_{0}}\left(k_{x}^{2}+k_{y}^{2}\right)$, where $m_{0}$ is the bare electron mass. At a fixed energy μ, the contour is a circle parameterized as $\left(k_{x},k_{y}\right)=\sqrt{2m_{0}\mu }\left(\cos\theta ,\sin\theta \right),\;\theta \in \left[0,2\pi \right)$. Using the method of characteristics, we get $\frac{d\theta }{ds}=-\frac{\mathrm{qB}}{m_{0}}$, or $\theta =\theta_{0}-\omega_{0}s$, where $\omega_{0}\equiv \frac{\mathrm{qB}}{m_{0}}$ is the cyclotron frequency of bare electrons. This leads to the periodicity in s to be $s_{0}=2\pi /\omega_{0}$, where we have chosen the clockwise trajectory such that $s_{0}>0$. The Fourier series of the velocity along the constant energy contour is given by $v_{x}\left(s\right)=\sqrt{\frac{\mu }{2m}}\left(e^{-i\omega_{0}s}+e^{i\omega_{0}s}\right)$, and $v_{y}\left(s\right)=\sqrt{\frac{\mu }{2m}}\frac{1}{i}\left(e^{-i\omega_{0}s}-e^{i\omega_{0}s}\right)$. Substituting into Eq. **21**, we obtain the conductivity tensor:

[22] $$\begin{array}{c} \sigma =q^{2}\tau \frac{\mu }{2\pi }\frac{1}{1+\omega_{0}^{2}\tau^{2}}\left(\begin{array}{cc} 1 & -\omega_{0}\tau \\ \omega_{0}\tau & 1 \end{array}\right). \end{array}$$

Note that the total number density of filled electrons is given by $n=\int \frac{d^{2}k}{{\left(2\pi \right)}^{2}}\Theta \left(\mu -\varepsilon_{k}\right)=\frac{m_{0}\mu }{2\pi }$. We therefore reproduce the well-known magnetoconductivity tensor:

[23] $$\begin{array}{c} \sigma =\frac{nq^{2}\tau }{m_{0}}\frac{1}{1+\omega_{0}^{2}\tau^{2}}\left(\begin{array}{cc} 1 & -\omega_{0}\tau \\ \omega_{0}\tau & 1 \end{array}\right). \end{array}$$

In this simple example of a closed FS, the longitudinal resistivity is given by $\frac{m_{0}}{nq^{2}\tau }$, independent of the magnetic field. The average of the velocity field, $v_{n=0}\equiv \frac{1}{s_{0}}\int_{0}^{s_{0}}dsv\left(s\right)$, vanishes. However, for an open FS, generally, $v_{n=0}\neq 0$, i.e., electrons have a finite drift velocity when a magnetic field causes them to traverse the contour (*SI Appendix*, Fig. S2). The impact of such a finite drift velocity on the magnetotransport can be qualitatively understood using the following example: In the expression for the conductivity tensor (Eq. **21**), we consider $v_{n=0}^{x}\neq 0$ but $v_{n=0}^{y}=0$. This corresponds to an open FS with a drift velocity along the x direction. In the high-field limit ( $\omega_{c}\tau \propto B\gg 1)$, we truncate the Fourier series at the leading order, yielding

[24] $$\begin{array}{c} \sigma_{\;\text{open FS}}\approx \frac{q^{3}B}{2\pi }\frac{\tau }{\omega_{c}}\left(\begin{array}{cc} {\left(v_{0}^{x}\right)}^{2} & -\frac{2\;\text{Im}(v_{-1}^{x}v_{1}^{y})}{\omega_{c}\tau }\\ \frac{2\;\text{Im}(v_{-1}^{x}v_{1}^{y})}{\omega_{c}\tau } & \frac{|v_{1}^{y}{|}^{2}}{\omega_{c}^{2}\tau^{2}} \end{array}\right), \end{array}$$

where we made use of the equality: $v_{-n}=v_{n}^{*}$. Inverting the matrix, we obtain the MR tensor:

[25] $$\begin{array}{c} \begin{array}{cc} \rho_{\;\text{open FS}}\approx & \;\frac{\left(2\pi \right)\omega_{c}}{q^{3}B\tau }\frac{1}{4\;\text{Im}{\left(v_{-1}^{x}v_{1}^{y}\right)}^{2}+{\left(v_{0}^{x}\right)}^{2}{\left|v_{1}^{y}\right|}^{2}}\\ & \times \left(\begin{array}{cc} |v_{1}^{y}{|}^{2} & 2\;\text{Im}(v_{-1}^{x}v_{1}^{y})\omega_{c}\tau \\ -2\;\text{Im}(v_{-1}^{x}v_{1}^{y})\omega_{c}\tau & {\left(v_{0}^{\left(x\right)}\right)}^{2}{\left(\omega_{c}\tau \right)}^{2} \end{array}\right). \end{array} \end{array}$$

We see that for an open FS, the longitudinal MR has nonsaturating $B^{2}$ behavior along the axis with a zero drift velocity ($\hat{y}$ in the above example), and saturating behavior (constant in B in this simple model) along the other axis.

## 3. Comparison between Theory and Experiment

As we will see, the theory satisfactorily explains the weak-field magnetotransport measurements presented in ref. 45. We then present two predictions of the theory that we did not anticipate prior to starting this work: the dependence of the principal axis of transport on filling, and the behavior of magnetoresistance and quantum oscillations at densities between the CNP and the onset of quadratic MR. The former has important implications, but it cannot be confirmed with our present datasets because of limitations of the Hall bar geometry. The latter can be considered smoking gun evidence for the presence of the lowest-energy van Hove point and the energetic splitting of the Dirac cones.

We present calculations for $\theta =1.38^{{}^{\circ}}$ (independently measured for the region of the experimental device we focus on), and ϵ=0.2% and $\varphi =0^{{}^{\circ}}$, parameters which are not measured in the experiment but are chosen to yield reasonable quantitative agreement between the theoretical and experimental results with respect to the filling range of open FSs and to the frequencies of magnetoresistance oscillations to be presented later. The general phenomena of open FSs and quadratic MR hold for a broad range of heterostrain parameters ϵ and φ. We do not perform fine-tuning of these input parameters for two reasons: 1. We do not expect our strained BM model in Eq. **2** to yield precise quantitative agreement with experiment. Specifically, the model has particle–hole symmetry, which is absent from experimental measurements. More sophisticated noninteracting model calculations (49, 50) as well as interaction renormalizations (61) would likely be necessary to properly account for such details. 2. Increasing ϵ broadens the filling range that displays open FSs, but for a given ϵ, varying φ strongly tunes this range (Fig. 1 *G* and *H*), so there is some flexibility in assigning the two parameters to match the experimental measurements. This might be overcome by also seeking to match the position of the lowest van Hove singularity and the evolution of sizes of the two Fermi pockets near the CNP, but the simplifications in the BM model noted in (1) above caution us against drawing strong quantitative conclusions from the comparison.

**Fig. 2.**
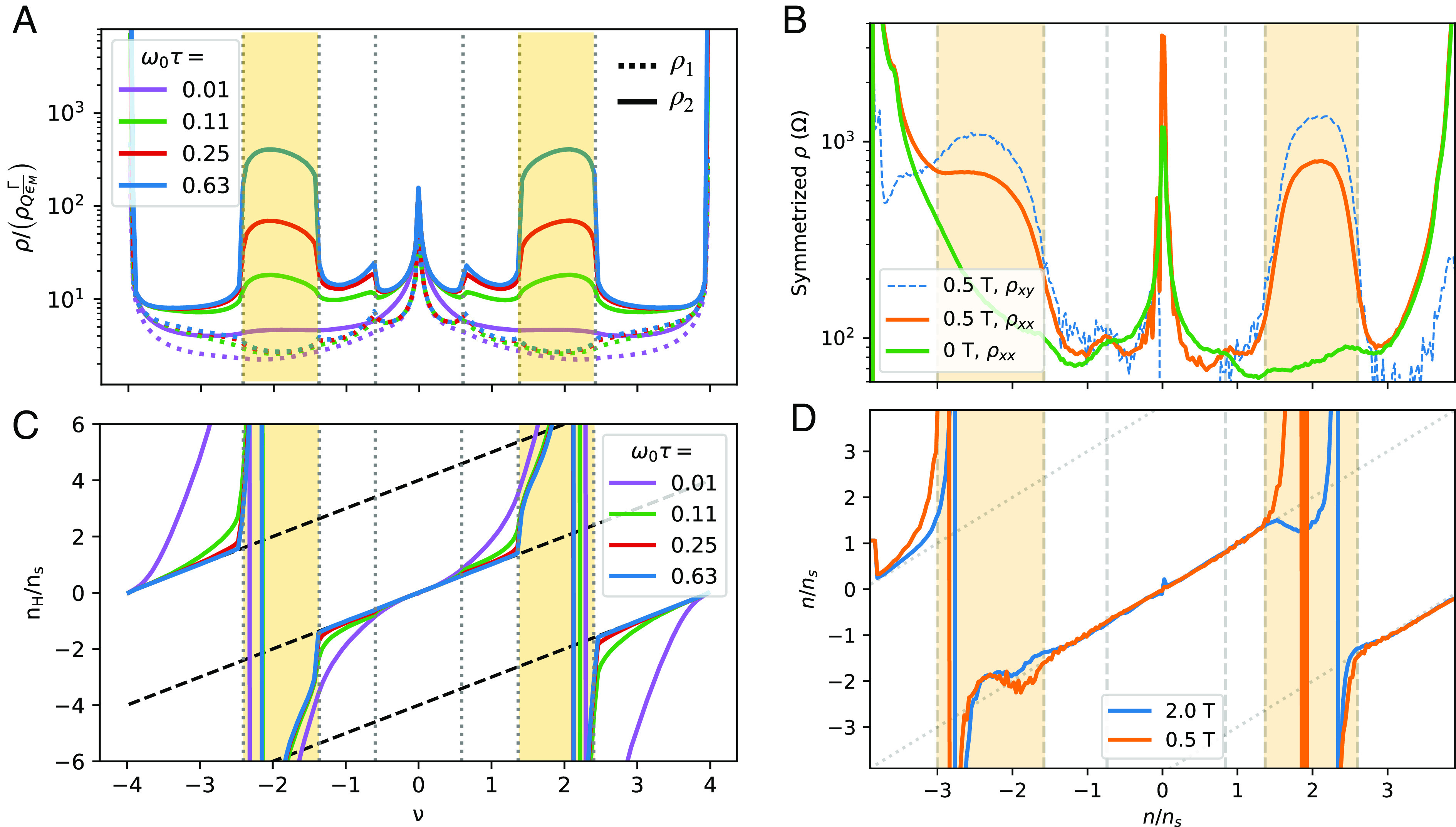
Magnetotransport properties of strained TBG. (*A* and *B*) Theoretical calculations of transport properties as a function of magnetic field strengths $\omega_{0}\tau$ for $\theta =1.38^{{}^{\circ}}$, ϵ=0.2% and $\varphi =0^{{}^{\circ}}$. The cyclotron frequency $\omega_{c}$ defined in Eq. **20** is filling dependent, hence our choice to use the bare cyclotron frequency $\omega_{0}\tau =eB\tau /m_{0}$. The vertical dashed lines mark the calculated van Hove points, with yellow regions indicating open FSs. (*A*) Longitudinal MR along the principal axes ${\hat{e}}_{1}$ (dashed) and ${\hat{e}}_{2}$ (solid) in units of $\rho_{Q}\frac{\Gamma }{\epsilon_{M}}$, where $\rho_{Q}\equiv h/e^{2}$ is the quantum of resistance, Γ≡ħ/τ is the transport decay rate, and $\epsilon_{M}\equiv \hbar v_{F}\left|K\right|\theta$ is the characteristic energy scale for moiré electrons. For a transport rate Γ=0.1meV, $\rho_{Q}\frac{\Gamma }{\epsilon_{M}}\approx 9.6\Omega$, and $\omega_{0}\tau \approx 0.13$ is equivalent to a magnetic field strength B≈0.11T. (*B*) Hall number $n_{H}\equiv B/e\rho_{H}$ plotted in units of $n_{s}$, where $2n_{s}$ is the total electron density for the narrow bands. (*C* and *D*) Experimental measurements of longitudinal MR (contact pair 14-15) and transverse MR (contact pair 15 - 5) for the TBG sample in ref. 45, which has 20 contacts, at 1.6 K. Vertical dashed lines mark the densities that we ascribe to van Hove points based on the cusp near ν∼0.8 and the onset of quadratic MR (shaded yellow). Finite-field resistivities in panel (*C*) are symmetrized: ρ=(ρ(B)+ρ(−B))/2. Panel (*D*) is calculated from the antisymmetrized transverse resistivity. In the experimental figures, $n/n_{s}$ labels the electron filling fractions per moiré unit cell.

In Fig. 2, we show the computed MR along the principal transport axes (*A*) and the Hall number (*B*). For comparison, we plot the experimentally measured longitudinal and transverse resistivities (*C*) and Hall number (*D*) for the TBG device studied in ref. 45.

In the filling ranges with open FSs, the calculated $\rho_{2}\left(B\right)$ exhibits quadratic nonsaturating MR, whereas $\rho_{1}\left(B\right)$ saturates. The filling range for which quadratic MR occurs is bounded by the two outermost van Hove points of the zero-field strained band structure. In experiment, we observe quadratic MR in longitudinal resistivity within a similar range of fillings. More strikingly, we observe quadratic MR in the transverse resistivity as well. In some cases, with increasing field, the symmetric part of the transverse resistivity becomes larger than that of the longitudinal resistivity. As discussed earlier, this degree of mixing can be attributed to the misalignment between the strain-induced but filling-dependent principal axis of transport and the direction of current flow in the Hall bar geometry.

At the first van Hove point (ν≈±0.6), the nonanalyticity in the density of states leads to a cusp in the first derivative of the zero-field resistivity with respect to filling (*SI Appendix*, Fig. S6). As shown in Fig. 2*A*, at B≠0 the longitudinal resistance as a function of filling develops a cusp at the first van Hove point. The cusp becomes more pronounced with increasing B. Experimentally, as shown in Fig. 2*C*, there is a cusp-like feature developing at |ν|∼0.5−0.8 depending on the contact pair within the device used, consistent with theoretical predictions. In many contact pairs, this feature presents as a shoulder at B=0, only developing into a cusp at B∼0.1 T (*SI Appendix*, Fig. S7).

As depicted in Fig. 2*B*, the calculated filling dependence of the Hall number shows two singular sign changes near ν≈±2, each falling within inside an open FS region. The filling at which each sign-changing singularity occurs is B-independent and is not directly associated with any van Hove point (see *SI Appendix*, Fig. S5 for a plot of $\rho_{H}\left(B\right)$, which crosses zero at the same filling fraction inside the open FS filling range for varying field strength). Moreover, the filling dependence of the Hall number $n_{H}$ tracks the filling fraction in a broad filling range near the CNP, with the filling range being extended upon increasing B. Note also that the Hall number is generally field-dependent due to the impact of crystalline symmetries on the band dispersions (See *SI Appendix*, section IV.A for a detailed analysis). In Fig. 2*D*, we observe the same general shape of the Hall number. Within the open FS filling range, however, the measured Hall number qualitatively deviates from the theoretical curves. We tentatively attribute this to a small constant offset in the magnetic field of order 10 to 20 mT, an amount typical for trapped flux in a NbTi/Nb${}_{3}$Sn superconducting magnet like ours. To explicate, in the open FS regime, we expect (and observe) a large quadratic symmetric component of the transverse resistivity together with a vanishing antisymmetric component. Hence, quantification of the antisymmetric component involves subtracting two large numbers. An offset of only a few mT in the assigned field will lead to a small part of the symmetric component mixing into the antisymmetric component, leading to these deviations from theory (*SI Appendix*, Fig. S8).

**Fig. 3.**
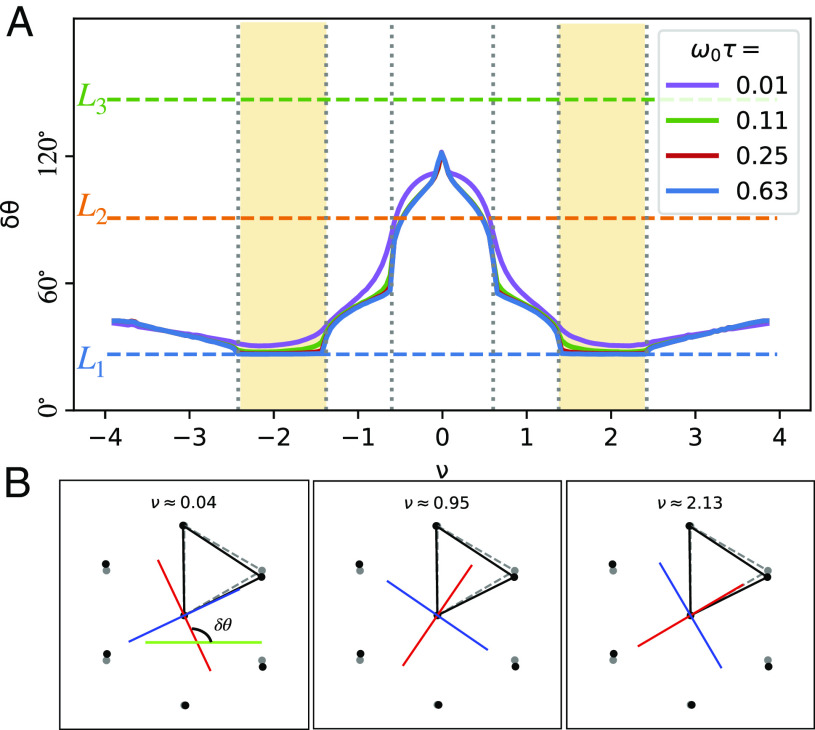
(*A*) Rotation of the transport principal axis ${\hat{e}}_{1}$ with respect to the global coordinate system for strained BM with ϵ=0.2% and $\varphi =0^{{}^{\circ}}$. The three horizontal dashed lines are the bond directions. In the open FS region, the saturating MR axis is locked to the shortest bond ($L_{1}$) direction. However, it rapidly rotates in the closed FS region upon approaching the CNP. (*B*) Principal transport axes ${\hat{e}}_{1}$ (red) and ${\hat{e}}_{2}$ (blue) for a few filling fractions. Near the CNP, ${\hat{e}}_{1}$ is perpendicular to the shortest moiré bond direction. In the open FS filling range (e.g., ν≈2.13), it is rotated to be parallel to the shortest bond direction.

Our calculation finds a dramatic rotation of the principal axis with filling, as illustrated in Fig. 3. In the filling range with open FSs, the principal axis with saturating MR (${\hat{e}}_{1}$) is aligned with direction of the shortest moiré triangular bond, suggesting that the electrons are hopping more efficiently along the shortest bond, which leads to a larger conductivity and therefore a smaller resistivity. Remarkably, when filling is changed from the second van Hove point (ν≈±1.3) to the vicinity of the CNP, ${\hat{e}}_{1}$ rotates by about 90°. This is likely associated with the larger Fermi pocket encircling a Dirac point being elongated in a direction nearly perpendicular to the open FS contours; see, for example, Fig. 1*D*–*F*. Such rotation of the principal transport axis with filling has been attributed to interaction-induced nematicity (14), but we can now see that in some contexts, it could occur purely due to strain-induced bandstructure effects. Such a filling-dependent rotation of the principal transport axis was not possible to observe in ref. 45 using the Hall bar geometry, where only $\rho_{\mathrm{xx}}$ and $\rho_{\mathrm{yx}}$ are measured but not $\rho_{\mathrm{yy}}$. Additional transport measurements are needed, where the filling dependence of the entire resistivity tensor can be mapped out.

**Fig. 4.**
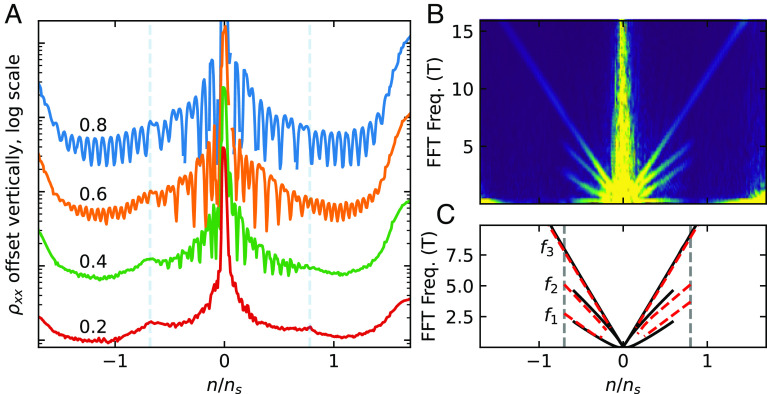
(*A*) Line cuts of MR near the CNP taken at 26 mK in contact pair 4 to 5 at the indicated field strengths, in Tesla. Vertical dashed lines indicate our estimated location of the lowest-energy van Hove points, based on the cusps in resistivity at low field. Within the region bounded by these points, the quantum oscillations show up before 0.4 T, and their relative strengths do not follow a simple pattern. Outside of this region, the quantum oscillations onset at higher field, and every multiple of 4 quantum Hall filling fraction is observed relatively equally. (*B*) Fourier transform of the quantum oscillation data with respect to 1/B. It reveals a transition from two pockets to one pocket at the lowest-energy van Hove points. (*C*) Schematic description of the frequencies observed in panel (*B*). Red dashed lines are frequencies from the experimental data. Solid black lines are predictions from the theory for ϵ=0.2% and $\varphi =0^{{}^{\circ}}$. The two frequencies $f_{1}$ and $f_{2}$ sum to the one-pocket frequency $f_{3}$ that extends beyond the first van Hove point. They additionally account for the nontrivial relative strengths of the quantum oscillations within the bounds of the first van Hove points. As with other details of this work, the theory predicts electron–hole symmetry, while some asymmetry is observed in experiment.

Since this theory predicts a third van Hove point between the CNP and the filling range with open FSs, a direct experimental signature of this van Hove point is desired. In Fig. 4, we reanalyze quantum oscillation measurements of the TBG device discussed in ref. 45. The effective cyclotron mass $m^{*}$ is light in the filling range with two small closed Fermi pockets and dramatically heavier in the filling range with only one closed pocket (Fig. 1*D*–*F* and *SI Appendix*, Fig. S5). The large difference in masses on either side of the innermost van Hove singularity can account for the substantially lower-field onset of quantum oscillations close to the CNP than away from it, as shown in Fig. 4*A*. Fig. 4*B* is a Fourier transform of the quantum oscillation data with respect to 1/B. In the filling range of −0.7≤ν≤0.8, three distinct frequencies $f_{i=1,2,3}$ are clearly observed in the data, with $f_{1}$ and $f_{2}$ corresponding to two small Fermi pockets, and $f_{3}=f_{1}+f_{2}$ to the breakdown orbit: By 1T, the inverse magnetic length is comparable to the momentum space distance between the two small Fermi pockets (67). Each edge of this filling range is marked by two features: 1. $f_{1}$ and $f_{2}$ disappear from the Fourier transform, leaving only $f_{3}$ at higher electron or hole filling. 2. A cusp-like feature occurs in longitudinal MR (Fig. 2 *A* and *C*). These both are predicted by our model as features of a Lifshitz transition on either side of the CNP, associated with crossing the lowest-energy van Hove points. This detailed match unambiguously demonstrates the existence of a third van Hove singularity to each side of the CNP, at electron or hole filling range (respectively) lower than the onset of $B^{2}$ MR. The Hall number does not show a sign-changing singularity at this van Hove point, as illustrated in Fig. 2 *B* and *D*.

The frequencies $f_{1,2}$ are a strong constraint on the amount of heterostrain in the TBG sample. Specifically, as illustrated in Fig. 4*C*, the frequency $f_{2}$ is roughly two times $f_{1}$, showing that the two small Fermi pockets have an area ratio ∼2:1. Theoretically, as illustrated by the solid black lines in Fig. 4*C*, for a heterostrain strength ϵ=0.2% and $\varphi =0^{{}^{\circ}}$, the areas $A_{i=1,2}$ of the two small pockets, when converted to frequency via $f_{i}^{-1}\equiv {\left(\Delta \frac{1}{B}\right)}_{i}=\frac{2\pi e}{\hbar A_{i}}$, are in good agreement with experiment. Furthermore, also note that the frequencies $f_{1,2}$ extrapolate to 0 at ν≈±0.04, showing that the two Dirac points are shifted to finite (opposite) filling fractions by heterostrain.

We observe behavior qualitatively similar in all respects to that in Fig. 4*A* in all 3 longitudinal contact pairs for which we have dilution-fridge measurements (*SI Appendix*, Fig. S11).

## 4. Summary and Outlook

In summary, we have shown that due to the large size of the moiré unit cell at small twist angles, even a small amount of uniaxial heterostrain on the microscopic scale can lead to dramatic changes in the narrow bands of twisted bilayer graphene. A key feature of the strained bandstructure is the splitting of the respective energetic degeneracies of the two Dirac points and the three van Hove points. The splitting of the two Dirac points leads to a semimetallic state with two small Fermi pockets at the CNP. On the other hand, the two outermost van Hove points bound a broad filling range near ν=±2 where the constant energy contours become open. Interestingly, the elongation of the larger Fermi pocket near the CNP is perpendicular to that of the open FSs, the latter being perpendicular to the direction of the shortest moiré triangular bond.

We have analyzed the resulting magnetotransport in strained TBG in the framework of the Boltzmann equation using the method of characteristics, treating the magnetic field nonperturbatively. We showed that a nonsaturating quadratic longitudinal magnetoresistance in a broad filling range near ν=±2 naturally arises due to the heterostrain-induced open Fermi surfaces, therefore explaining in remarkable detail experimental results in off-magic-angle devices with lattice anisotropy (45). We have also shown that the sign-changing singularities in the Hall number occur in the open FS filling range and are not directly associated with any van Hove singularity as commonly assumed, e.g., in ref. 68. Furthermore, our theory reveals a dramatic rotation of the transport principal axis as the filling is tuned from the charge neutrality point to the filling range of open Fermi surfaces, without invoking interaction-induced electronic nematicity.

Given the importance of energy-shifted van Hove points in the transport properties of TBG devices, we have analyzed previous quantum oscillation data, which has revealed a Lifshitz transition from two pockets to one pocket at a filling fraction where the innermost van Hove singularity is predicted to occur based on theoretical calculations, offering strong evidence of heterostrain effects on these devices. We have further proposed several additional signatures to look for in future experiments. These include a significant difference in cyclotron mass on either side of the innermost van Hove singularity (probed qualitatively but not quantitatively in the extant experiment) and a principal transport axis with saturating magnetoresistance in the open Fermi surface filling range.

Finally, given the amplifying effect of a small strain at the underlying carbon lattice scale on the moiré lattice scale, the latter of which controls the electronic behavior within the narrow bands, it is tantalizing to consider strain engineering of such devices to achieve effects which in regular solids would require applying strain magnitudes incompatible with structural stability.

## Supplementary Material

Appendix 01 (PDF)

## Data Availability

The data for Fig. 4 and *SI Appendix*, Fig. S11 were previously published with ref. 45 in ref. 69. All other experimental data are available at ref. 70. The code for calculating magnetotransport of TBG under uniaxial heterostrain is available at ref. 71. Tabular data with analysis notebook data have been deposited in Stanford Digital Repository (https://doi.org/10.25740/zs335dw3715).

## Footnotes

^*^We checked numerically that adding a small homogeneous strain in addition to a heterostrain of similar strength yields band and transport properties almost identical to those produced by adding a heterostrain alone.

## References

[1] Y. Cao , Correlated insulator behaviour at half-filling in magic-angle graphene superlattices. Nature **556**, 80–84 (2018). 29512654 10.1038/nature26154

[2] Y. Cao , Unconventional superconductivity in magic-angle graphene superlattices. Nature **556**, 43–50 (2018). 29512651 10.1038/nature26160

[3] A. Kerelsky , Maximized electron interactions at the magic angle in twisted bilayer graphene. Nature **572**, 95–100 (2019). 31367030 10.1038/s41586-019-1431-9

[4] X. Lu , Superconductors, orbital magnets and correlated states in magic-angle bilayer graphene. Nature **574**, 653–657 (2019). 31666722 10.1038/s41586-019-1695-0

[5] Y. Jiang , Charge order and broken rotational symmetry in magic-angle twisted bilayer graphene. Nature **573**, 91–95 (2019). 31365921 10.1038/s41586-019-1460-4

[6] M. Yankowitz , Tuning superconductivity in twisted bilayer graphene. Science **363**, 1059–1064 (2019). 30679385 10.1126/science.aav1910

[7] Y. Choi , Electronic correlations in twisted bilayer graphene near the magic angle. Nat. Phys. **15**, 1174–1180 (2019).

[8] A. L. Sharpe , Emergent ferromagnetism near three-quarters filling in twisted bilayer graphene. Science **365**, 605–608 (2019). 31346139 10.1126/science.aaw3780

[9] Y. Xie , Spectroscopic signatures of many-body correlations in magic-angle twisted bilayer graphene. Nature **572**, 101–105 (2019). 31367031 10.1038/s41586-019-1422-x

[10] U. Zondiner , Cascade of phase transitions and dirac revivals in magic-angle graphene. Nature **582**, 203–208 (2020). 32528091 10.1038/s41586-020-2373-y

[11] D. Wong , Cascade of electronic transitions in magic-angle twisted bilayer graphene. Nature **582**, 198–202 (2020). 32528095 10.1038/s41586-020-2339-0

[12] M. Serlin , Intrinsic quantized anomalous hall effect in a moiré heterostructure. Science **367**, 900–903 (2020). 31857492 10.1126/science.aay5533

[13] P. Stepanov , Untying the insulating and superconducting orders in magic-angle graphene. Nature **583**, 375–378 (2020). 32632215 10.1038/s41586-020-2459-6

[14] Y. Cao , Nematicity and competing orders in superconducting magic-angle graphene. Science **372**, 264–271 (2021). 33859029 10.1126/science.abc2836

[15] X. Liu , Tuning electron correlation in magic-angle twisted bilayer graphene using coulomb screening. Science **371**, 1261–1265 (2021). 33737488 10.1126/science.abb8754

[16] A. T. Pierce , Unconventional sequence of correlated Chern insulators in magic-angle twisted bilayer graphene. Nat. Phys. **17**, 1210–1215 (2021).

[17] S. Wu, Z. Zhang, K. Watanabe, T. Taniguchi, E. Y. Andrei, Chern insulators, van Hove singularities and topological flat bands in magic-angle twisted bilayer graphene. Nat. Mater. **20**, 488–494 (2021). 33589799 10.1038/s41563-020-00911-2

[18] J. Yu , Correlated Hofstadter spectrum and flavour phase diagram in magic-angle twisted bilayer graphene. Nat. Phys. **18**, 825–831 (2022).

[19] R. Bistritzer, A. H. MacDonald, Moiré bands in twisted double-layer graphene. Proc. Natl. Acad. Sci. U.S.A. **108**, 12233–12237 (2011). 21730173 10.1073/pnas.1108174108 PMC3145708

[20] E. Y. Andrei, A. H. MacDonald, Graphene bilayers with a twist. Nat. Mater. **19**, 1265–1275 (2020). 33208935 10.1038/s41563-020-00840-0

[21] L. Balents, C. R. Dean, D. K. Efetov, A. F. Young, Superconductivity and strong correlations in moiré flat bands. Nat. Phys. **16**, 725–733 (2020).

[22] M. Koshino , Maximally localized Wannier orbitals and the extended Hubbard model for twisted bilayer graphene. Phys. Rev. X **8**, 031087 (2018).

[23] H. C. Po, L. Zou, A. Vishwanath, T. Senthil, Origin of Mott insulating behavior and superconductivity in twisted bilayer graphene. Phys. Rev. X **8**, 031089 (2018).

[24] J. Kang, O. Vafek, Symmetry, maximally localized Wannier states, and a low-energy model for twisted bilayer graphene narrow bands. Phys. Rev. X **8**, 031088 (2018).

[25] J. Kang, O. Vafek, Strong coupling phases of partially filled twisted bilayer graphene narrow bands. Phys. Rev. Lett. **122**, 246401 (2019). 31322361 10.1103/PhysRevLett.122.246401

[26] Z. Song , All magic angles in twisted bilayer graphene are topological. Phys. Rev. Lett. **123**, 036401 (2019). 31386469 10.1103/PhysRevLett.123.036401

[27] M. Xie, A. H. MacDonald, Nature of the correlated insulator states in twisted bilayer graphene. Phys. Rev. Lett. **124**, 097601 (2020). 32202880 10.1103/PhysRevLett.124.097601

[28] N. Bultinck , Ground state and hidden symmetry of magic-angle graphene at even integer filling. Phys. Rev. X **10**, 031034 (2020).

[29] Y. Zhang, K. Jiang, Z. Wang, F. Zhang, Correlated insulating phases of twisted bilayer graphene at commensurate filling fractions: A Hartree-Fock study. Phys. Rev. B **102**, 035136 (2020).

[30] T. Cea, F. Guinea, Band structure and insulating states driven by coulomb interaction in twisted bilayer graphene. Phys. Rev. B **102**, 045107 (2020).

[31] J. Kang, O. Vafek, Non-abelian dirac node braiding and near-degeneracy of correlated phases at odd integer filling in magic-angle twisted bilayer graphene. Phys. Rev. B **102**, 035161 (2020).

[32] O. Vafek, J. Kang, Renormalization group study of hidden symmetry in twisted bilayer graphene with coulomb interactions. Phys. Rev. Lett. **125**, 257602 (2020). 33416368 10.1103/PhysRevLett.125.257602

[33] B. Lian , Twisted bilayer graphene. IV. Exact insulator ground states and phase diagram. Phys. Rev. B **103**, 205414 (2021).

[34] B. A. Bernevig , Twisted bilayer graphene. V. Exact analytic many-body excitations in coulomb Hamiltonians: Charge gap, goldstone modes, and absence of cooper pairing. Phys. Rev. B **103**, 205415 (2021).

[35] F. Xie , Twisted bilayer graphene. VI. An exact diagonalization study at nonzero integer filling. Phys. Rev. B **103**, 205416 (2021).

[36] P. Potasz, M. Xie, A. H. MacDonald, Exact diagonalization for magic-angle twisted bilayer graphene. Phys. Rev. Lett. **127**, 147203 (2021). 34652208 10.1103/PhysRevLett.127.147203

[37] Y. H. Kwan , Kekulé spiral order at all nonzero integer fillings in twisted bilayer graphene. Phys. Rev. X **11**, 041063 (2021).

[38] D. E. Parker, T. Soejima, J. Hauschild, M. P. Zaletel, N. Bultinck, Strain-induced quantum phase transitions in magic-angle graphene. Phys. Rev. Lett. **127**, 027601 (2021). 34296891 10.1103/PhysRevLett.127.027601

[39] L. Huder , Electronic spectrum of twisted graphene layers under heterostrain. Phys. Rev. Lett. **120**, 156405 (2018). 29756887 10.1103/PhysRevLett.120.156405

[40] Z. Bi, N. F. Q. Yuan, L. Fu, Designing flat bands by strain. Phys. Rev. B **100**, 035448 (2019).

[41] F. Mesple , Heterostrain determines flat bands in magic-angle twisted graphene layers. Phys. Rev. Lett. **127**, 126405 (2021). 34597066 10.1103/PhysRevLett.127.126405

[42] N. P. Kazmierczak , Strain fields in twisted bilayer graphene. Nat. Mater. **20**, 956–963 (2021). 33859383 10.1038/s41563-021-00973-w

[43] K. Kim , van der Waals heterostructures with high accuracy rotational alignment. Nano Lett. **16**, 1989–1995 (2016). 26859527 10.1021/acs.nanolett.5b05263

[44] Y. Cao , Superlattice-induced insulating states and valley-protected orbits in twisted bilayer graphene. Phys. Rev. Lett. **117** (2016). 10.1103/PhysRevLett.117.116804 27661712

[45] J. Finney , Unusual magnetotransport in twisted bilayer graphene. Proc. Natl. Acad. Sci. U.S.A. **119**, e2118482119 (2022). 35412918 10.1073/pnas.2118482119 PMC9169859

[46] H. Suzuura, T. Ando, Phonons and electron-phonon scattering in carbon nanotubes. Phys. Rev. B **65**, 235412 (2002).

[47] N. N. T. Nam, M. Koshino, Lattice relaxation and energy band modulation in twisted bilayer graphene. Phys. Rev. B **96**, 075311 (2017).

[48] C. Kittel, Quantum Theory of Solids (Wiley, New York, 1963).

[49] O. Vafek, J. Kang, Continuum effective Hamiltonian for graphene bilayers for an arbitrary smooth lattice deformation from microscopic theories (2022).

[50] J. Kang, O. Vafek, Pseudo-magnetic fields, particle-hole asymmetry, and microscopic effective continuum Hamitonians of twisted bilayer graphene (2022).

[51] E. H. Hwang, S. Das Sarma, Acoustic phonon scattering limited carrier mobility in two-dimensional extrinsic graphene. Phys. Rev. B **77**, 115449 (2008).

[52] D. K. Efetov, P. Kim, Controlling electron-phonon interactions in graphene at ultrahigh carrier densities. Phys. Rev. Lett. **105**, 256805 (2010). 21231611 10.1103/PhysRevLett.105.256805

[53] K. Kaasbjerg, K. S. Thygesen, K. W. Jacobsen, Unraveling the acoustic electron-phonon interaction in graphene. Phys. Rev. B **85**, 165440 (2012).

[54] D. Grassano , Work function, deformation potential, and collapse of Landau levels in strained graphene and silicene. Phys. Rev. B **101**, 245115 (2020).

[55] J. M. B. Lopes, N. M. R. dos Santos, Peres, A. H. Castro Neto, Graphene bilayer with a twist: Electronic structure. Phys. Rev. Lett. **99**, 256802 (2007). 18233543 10.1103/PhysRevLett.99.256802

[56] B. A. Bernevig, Z. D. Song, N. Regnault, B. Lian, Twisted bilayer graphene. I. Matrix elements, approximations, perturbation theory, and a *k* · *p* two-band model. Phys. Rev. B **103**, 205411 (2021).

[57] J. Kang, B. A. Bernevig, O. Vafek, Cascades between light and heavy fermions in the normal state of magic-angle twisted bilayer graphene. Phys. Rev. Lett. **127**, 266402 (2021). 35029496 10.1103/PhysRevLett.127.266402

[58] F. Guinea, N. R. Walet, Electrostatic effects, band distortions, and superconductivity in twisted graphene bilayers. Proc. Natl. Acad. Sci. U.S.A. **115**, 13174–13179 (2018). 30538203 10.1073/pnas.1810947115 PMC6310832

[59] L. Rademaker, D. A. Abanin, P. Mellado, Charge smoothening and band flattening due to Hartree corrections in twisted bilayer graphene. Phys. Rev. B **100**, 205114 (2019).

[60] Z. A. H. Goodwin, V. Vitale, X. Liang, A. A. Mostofi, J. Lischner, Hartree theory calculations of quasiparticle properties in twisted bilayer graphene. Elect. Struct. **2**, 034001 (2020).

[61] Y. Choi , Interaction-driven band flattening and correlated phases in twisted bilayer graphene. Nat. Phys. **17**, 1375–1381 (2021).

[62] M. Audin, M. Damian, *Morse Theory and Floer Homology* (Springer, 2014).

[63] M. O. Goerbig, J. N. Fuchs, G. Montambaux, F. Piéchon, Tilted anisotropic dirac cones in quinoid-type graphene and *α* -${\left(\;\text{BEDT-TTF}\right)}_{2}{\;\text{i}}_{3}$. Phys. Rev. B **78**, 045415 (2008).

[64] A. A. Soluyanov , Type-ii Weyl semimetals. Nature **527**, 495–498 (2015). 26607545 10.1038/nature15768

[65] M. Xie, A. H. MacDonald, Weak-field hall resistivity and spin-valley flavor symmetry breaking in magic-angle twisted bilayer graphene. Phys. Rev. Lett. **127**, 196401 (2021). 34797159 10.1103/PhysRevLett.127.196401

[66] I. Lifshits, M. Azbel, M. Kaganov, *Electron Theory of Metals* (Springer, 1973).

[67] D. Schoenberg, Magnetic Oscillations in Metals (2009).

[68] J. M. Park, Y. Cao, K. Watanabe, T. Taniguchi, P. Jarillo-Herrero, Tunable strongly coupled superconductivity in magic-angle twisted trilayer graphene. Nature **590**, 249–255 (2021). 33526935 10.1038/s41586-021-03192-0

[69] J. Finney , Data for: Unusual magnetotransport in twisted bilayer graphene, Stanford Digital Repository (2021). 10.25740/tm725vs8229.

[70] X. Wang , Data for: Unusual magnetotransport in twisted bilayer graphene from strain-induced open fermi surfaces, Stanford Digital Repository (2023). 10.25740/zs335dw3715. PMC10450440 37579169

[71] X. Wang , Code for: Unusual magnetotransport in twisted bilayer graphene from strain-induced open fermi surfaces (2033). https://github.com/xywang2017/TBG_MR. 10.1073/pnas.2307151120 PMC10450440 37579169

