# Supplementary material for: Unusual magnetotransport in twisted bilayer graphene from strain-induced open Fermi surfaces
Source: Proc Natl Acad Sci U S A. 2023 Aug 14;120(34):e2307151120. doi: 10.1073/pnas.2307151120 (PMC10450440; doi:10.1073/pnas.2307151120)
Supplement: Supplementary file 1 — Appendix 01 (PDF) [file pnas.2307151120.sapp.pdf]

Supplementary Materials for "Unusual magnetoresistance in twisted bilayer graphene from strain induced open Fermi surfaces"

Xiaoyu Wang,[1,*] Joe Finney,[2,3,*] Aaron Sharpe,[4] Linsey Rodenbach,[2,3] Connie Hsueh,[5,3] Kenji Watanabe,[6] Takashi Taniguchi,[7] Marc Kastner,[2,3,8,†] Oskar Vafek,[1,9,‡] and David Goldhaber-Gordon[2,3,§]

[1]*National High Magnetic Field Laboratory, Tallahassee, Florida, 32310, USA*
[2]*Department of Physics, Stanford University, Stanford, CA 94305*
[3]*Stanford Institute for Materials and Energy Sciences, SLAC National Accelerator Laboratory, Menlo Park, CA 94025*
[4]*Materials Physics Department, Sandia National Laboratories, Livermore, CA, USA*
[5]*Department of Applied Physics, Stanford University, Stanford, CA 94305*
[6]*Research Center for Functional Materials, National Institute for Materials Science, 1-1 Namiki, Tsukuba 305-0044, Japan*
[7]*International Center for Materials Nanoarchitectonics, National Institute for Materials Science, 1-1 Namiki, Tsukuba 305-0044, Japan*
[8]*Department of Physics, Massachusetts Institute of Technology, Cambridge, MA 02139*
[9]*Department of Physics, Florida State University, Tallahassee, Florida 32306, USA*

We present additional theoretical results and experimental measurements in support of the main text.

I. HETEROSTRAIN EFFECTS ON THE GEOMETRY OF MOIRÉ SUPERLATTICE

Our off-magic-angle twisted bilayer graphene (TBG) devices in Ref. [1] are prepared using the "tear-and-stack" procedure, and as a result, strain is inevitably introduced. Here we first show that while the moiré unit cell vectors are strongly deformed by even an infinitesimal amount of uniaxial heterostrain in the device, the unit cell area is much less affected. As a result, for the off-magic-angle device studied in Ref. [1], we can have a good estimate of the twist angle (θ) based on the moiré unit cell area alone.

In the limit of small deformations, both the uniaxial heterostrain and a small twist angle are captured via a coordinate transformation: $\mathbf{r}'_l = \mathbf{r} + \mathbf{u}_l(\mathbf{r})$, where $l = t, b$ labels the top (bottom) graphene layers, and $\mathbf{u}_l(\mathbf{r}) \approx \mathcal{E}_l \mathbf{r}$ is the local deformation field. The symmetric and antisymmetric part of the 2×2 tensor $\mathcal{E}_l$ describes strain and rotation respectively. For twist angle (θ) and a uniaxial heterostrain of strength (ϵ) and direction (φ), we parameterize $\mathcal{E}_t = -\mathcal{E}_b \equiv \mathcal{E}/2$, where $\mathcal{E} \equiv \mathcal{T}(\theta) + \mathcal{S}(\epsilon, \varphi)$, and given by:

$$\mathcal{T}(\theta) = \begin{pmatrix} 0 & -\theta \\ \theta & 0 \end{pmatrix}, \ \mathcal{S}(\epsilon, \varphi) = R_\varphi^T \begin{pmatrix} -\epsilon & 0 \\ 0 & \nu\epsilon \end{pmatrix} R_\varphi. \tag{1}$$

Here R_φ is the two-dimensional rotation matrix, and $\nu \approx 0.16$ is the Poisson ratio [2]. Physically, $\epsilon > 0$ corresponds to compressing the top layer while streching the bottom layer along the x-axis. A relative deformation $\mathcal{E}$ between the graphene bilayers generate a moiré superlattice, with moiré reciprocal lattice vectors given by:

$$\mathbf{g}_{i=1,2} = \mathcal{E}^T \mathbf{G}_{i=1,2}, \tag{2}$$

where $\mathbf{G}_i$ are reciprocal lattice vectors of the undeformed monolayer graphene. Eq. (2) can be used to uniquely determine the three parameters $(\theta, \epsilon, \varphi)$. Additionally it also determines a global angle α that measures the rotation between the lab and theoretical coordinate systems.

Uniaxial heterostrain has a dramatic effect on the distortion of the moiré unit cell vectors, as $|\delta\mathbf{g}|/|\mathbf{g}| \sim \mathcal{O}(\epsilon/\theta)$. However, its effect on the moiré unit cell area is much smaller. To show this, note that the area of the moiré Brillouin zone is calculated as:

$$A_{mBZ} = |(\mathbf{g}_1 \times \mathbf{g}_2) \cdot \hat{z}| = \left|\mathbf{g}_1^T (i\sigma_y) \mathbf{g}_2\right|, \tag{3}$$

where on the second equality we have used a vector notation $\mathbf{g}_i \equiv (g_{i,x}, g_{i,y})^T$. Following Eq. (2), we obtain that the area of the moiré Brillouin zone is independent on φ, and calculated as:

$$A_{mBZ} = (\theta^2 - \nu^2\epsilon^2) A_{BZ}, \tag{4}$$

* These two authors contributed equally
† mkastner@mit.edu
‡ vafek@magnet.fsu.edu
§ goldhaber-gordon@stanford.edu

where $A_{BZ} \equiv |(\mathbf{G}_1 \times \mathbf{G}_2) \cdot \hat{z}|$ is the Brillouin zone area of the undeformed monolayer graphene. The area of the strained moiré unit cell can be calculated in a similar manner, and we get: $A_{m.u.c.} = A_{u.c.}/(\theta^2 - \nu^2\epsilon^2)$, where $A_{u.c.}$ is the unit cell area of undeformed monolayer graphene. Observe that the heterostrain only affects the area of the moiré unit cell by $\mathcal{O}(\nu^2\epsilon^2/\theta^2)$ which is much smaller than the linear distortion of moiré unit cell vectors.

With only a knowledge of the moiré unit cell areas in Ref. [1] (see Table I), we estimate the twist angle to be $\theta \sim 1.35° - 1.39°$ for various contact pairs studied using the Hall bar geometry.

II. CONSTRAINING HETEROSTRAIN FROM TRANSPORT MEASUREMENTS

contact pairs	unit cell area (nm^2) ±0.1	ν_1 ±0.05	ν_2 ±0.05	ν_3 ±0.05	ν_4 ±0.05	ν_5 ±0.05	ν_6 ±0.05
4 - 5	95.0	-2.95	-1.58	-0.74	0.84	1.47	2.42
5 - 6	91.5	-3.28	-1.75	-0.66	0.76	1.53	2.84
6 - 7	89.2	-3.70	-1.68	-0.39	0.50	1.57	2.92
7 - 8	91.8	-3.27	-1.63	-0.49	0.60	1.42	2.94
14 - 15	93.6	-3.10	-1.60	-0.75	0.85	1.50	2.46
15 - 16	90.5	-3.32	-1.66	-0.66	0.83	1.66	2.65
16 - 17	90.1	-3.33	-1.89	-0.44	0.50	1.78	2.89
17 - 18	91.8	-3.38	-1.63	-0.49	0.54	1.53	2.94

TABLE I. Table of moiré unit cell areas and filling fractions $\nu_{i=1...6}$ of the six van Hove singularities for different contact pairs of the Hall bar measurements in Ref. [1]. The filling fractions are obtained by-eye based on magnetotransport measurements (Fig. 9). Theoretical calculations predict non-analytic behaviors of longitudinal magnetoresistance at all van Hove singularities.

For the TBG device studied in Ref. [1], the deformed moiré lattice vectors were not measured. Nevertheless, here we show that magnetotransport measurements, along with theoretical calculations based on the strained Bistrizer-MacDonald (BM) Hamiltonian, offer strong constraints on the heterostrain in the device. We caution, however, that since the strained BM model is an approximate description of the narrow bands of TBG, a precise determination of heterostrain from model calculations is not feasible.

First of all, as predicted by theoretical calculations, the van Hove singularities of the band structure lead to non-analytic behavior for the longitudinal magnetoresistance as a function of electron filling. The filling fractions for the six van Hove singularities in the narrow band are listed in Table I for various contact pairs. Secondly, magnetic oscillations show a Lifshitz transition at the inntermost van Hove singularities (ν_3, ν_4), from two small Fermi pockets closer to the charge neutrality point to one Fermi pocket away from it. Furthermore, the areas of the two small Fermi pockets, as revealed by the frequencies of magnetic oscillations, show a $2:1$ or smaller ratio. Both the filling fractions for van Hove singularities and the pocket area size offer strong constraints for the heterostrain. Qualitatively, on the one hand, a broader filling range of open Fermi surfaces can be achieved by increasing the strength of uniaxial heterostrain. On the other hand, to obtain Fermi pocket area sizes near $2:1$ ratio or smaller, a smaller heterostrain is necessary as it leads to a weaker splitting of the two Dirac cones. For theoretical calculations presented in the main text, we find $\epsilon = 0.2\%$ and $\varphi = 0°$ to give reasonably good agreements with both experimental observations described above. A larger heterostrain strength ($\epsilon = 0.3\%$) will lead to a much larger pocket area ratio ($4:1$ for $\epsilon = 0.3\%$ and $\varphi = 0°$), inconsistent with magnetic oscillation measurements. On the other hand, a smaller heterostrain strength $\epsilon = 0.1\%$ decreases the filling range of open Fermi surfaces dramatically, inconsistent with the longitudinal magnetoresistance measurements.

III. DETAILED BAND STRUCTURE ANALYSIS FOR VARYING UNIAXIAL HETEROSTRAIN

In the main text we discussed the band structure of the strained TBG for $\epsilon = 0.2\%$. The main effect of uniaxial heterostrain is to break the respective energetic degeneracies of the two Dirac points and three van Hove points of a given band, therefore giving rise to a semimetallic state at charge neutrality point, and open Fermi surface regions bounded by the two outermost van Hove points. However for a larger heterostrain, the innermost van Hove point moves closer to one of the Dirac point. As a result, both Dirac cones become type II titled, and the innermost van Hove points of both the upper and lower bands are annihilated. In turn two new band extrema are formed. This is illustrated in Fig. 1.

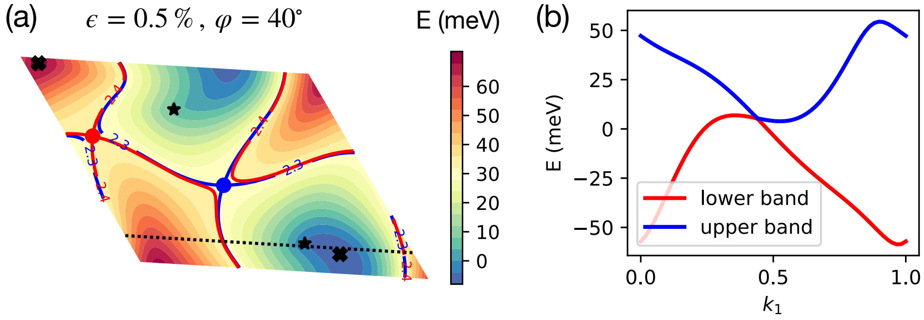

FIG. 1. Type II Dirac cone can occur at larger strengths of heterostain. Here we show an example of a type II Dirac cone for $\epsilon = 0.5\%$ and $\varphi = 40^\circ$. (a) is the energy contour map of the upper band in graphene valley $\mathbf{K}$, and (b) is the line cut corresponding to the black dotted line in (a).

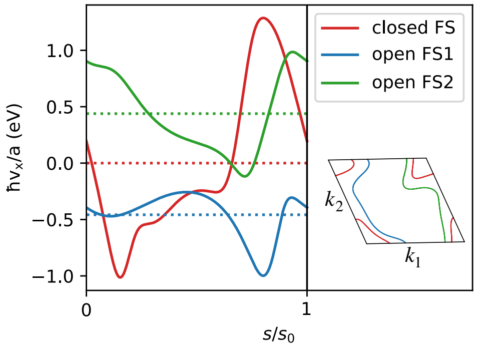

FIG. 2. Velocity field of typical closed and open Fermi surfaces. Whereas for a closed Fermi surface the averaged velocity vanishes, for open Fermi surfaces this is generally violated.

We also explore the possibilities of heterostrain-induced higher order van Hove singularities which is possible for the magic-angle TBG as discussed in Ref. [3]. We checked that for $\theta = 1.38^\circ$, and up to uniaxial heterostrain strength of $\epsilon = 0.7\%$, no higher order van Hove singularities are found. This shows that the band flattening effect at the magic angle may be important for strain engineering of higher order van Hove points.

In Fig. 2 we plot the velocity fields $v_x(s)$ and $v_y(s)$ on typical open and closed Fermi surfaces for the strained TBG, parameterized by $s \in [0, s_0)$ as defined in the main text. For the closed Fermi surface contours, the averaged velocity, $\mathbf{v}_{n=0} \equiv \frac{1}{s_0}\int_0^{s_0} \mathrm{d}s\mathbf{v}(s)$, is zero. On the other hand, for a typical open Fermi surface contour, it is finite, and as a result the electron traversing the open Fermi surface contour in the presence of a magnetic field has a finite drift velocity. As discussed in the main text, this is the reason for the non-saturating B^2 magnetoresistivity (MR) observed in strained TBG devices.

IV. MORE DETAILS ON MAGNETOTRANSPORT IN TBG

Here we show that while B^2 longitudinal MR generally occurs for strained TBG due to open Fermi surfaces, it does not occur for unstrained devices. In Fig. 3, the longitudinal MRs ρ_{xx} and ρ_{yy} as well as the Hall number n_H are plotted for an unstrained BM model calculation. First of all, $\rho_{xx} = \rho_{yy}$ since the unstrained TBG has C_{3z} rotational symmetry. Secondly, cusp-like features develop at the triply-degenerate van Hove point at filling fractions $\nu \approx \pm 1.4$, and are attributed to the non-analyticities in the density of states behavior at the van Hove singularities. Finally, unstrained TBG has saturating MR across all filling range, as illustrated in the inset to Fig. 3(a).

In Fig. 4 we show that for the globally defined coordinate system which is misaligned from the principal transport axis, the B^2 behavior generally dominates the MR, and therefore will show up in both ρ_{xx} and ρ_{yy} measurements. This remains true for a generic misalignment between the transport axis from experiment and the principal transport axis.

In Fig. 5 we show the Hall resistivity $\rho_H(B)$ for varying magnetic field strength. Since $\rho_H = B/n_H q$, wherever

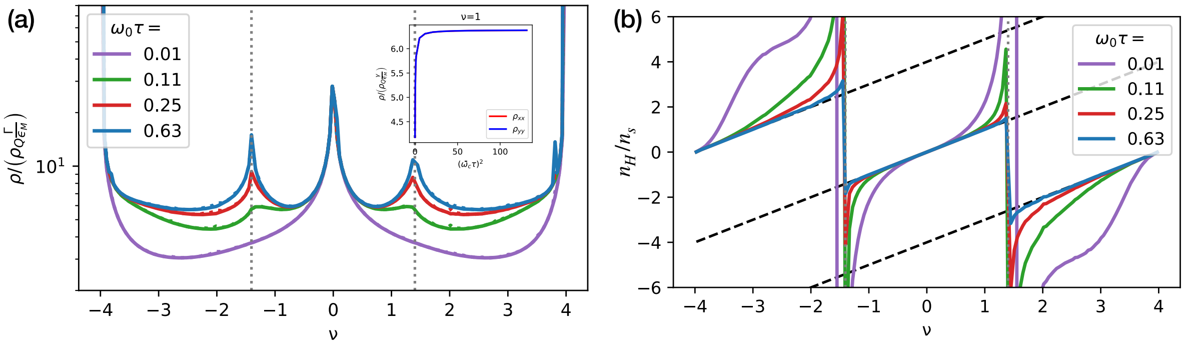

FIG. 3. (a) Longitudinal resistivitities ρ_{xx} (dashed) and ρ_{yy} (solid) for unstrained BM model as a function of filling ν. Different colors represent varying magnetic field strength. The inset shows a saturating MR as magnetic field is increased. (b) Hall number as a function of filling fraction. The magnetic field increases from blue to red. Gray dotted vertical lines mark positions of the van Hove singularities.

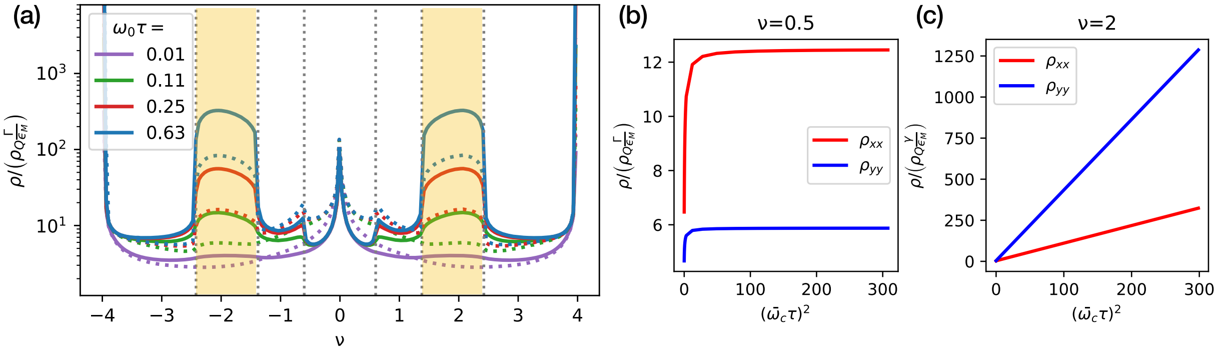

FIG. 4. (a) Longitudinal MR ρ_{xx} (dashed) and ρ_{yy} (solid) for strained BM with $\epsilon = 0.2\%$ and $\varphi = 0°$. Different colors represent varying magnetic field strength. Vertical dashed lines are positions of the van Hove points. Shaded areas are open Fermi surface regions. (b) In the closed Fermi surface region, MR saturates at large magnetic fields. (c) In the open Fermi surface region, MR exhibit non-saturating B^2 dependence along both directions.

$\rho_H(B)$ crosses zero and changes sign, the Hall number displays a sign-changing signularity. Fig. 5 clearly shows that $\rho_H(B)$ crosses zero in the open Fermi surface regions on both sides of the charge neutrality point, and independent on the strength of the B-field.

In Fig. 6 we illustrate the filling-dependent inverse cyclotron mass $1/m^*$ for strained BM model with $\epsilon = 0.2\%$ and $\varphi = 0°$. This is to highlight the dichotomy of light-heavy masses on either side of the innermost van Hove singularities closest to the charge neutrality point. This is consistent with the experimental observation of a much earlier onset field of quantum oscillations in filling range below the innermost van Hove point than above.

In Fig. 7 we also show the filling dependence of the $B = 0$ longitudinal resistivities and their derivatives with respect to filling. A key highlight is that the non-analyticities in the density of states at the van Hove points lead to kink-like features in the derivatives, but nearly invisible in the resistitivies themselves.

A. Magnetic field dependence of Hall number

As shown in both Fig. 3(b) and Fig. 2(b) in the main text, in the weak field limit ($\omega_c\tau \ll 1$), the Hall number n_H is dependent on the magnetic field. This differs from expectations from a parabolic band dispersion, where n_H is a constant reflecting the charge carrier density. Here we show that the magnetic field dependence of n_H generally comes from the higher harmonic terms of the Fermi velocities $\mathbf{v}(s) = \sum_n \mathbf{v}_n e^{in2\pi s/s_0}$ [cf. Eq. (21) of the main text] in the presence of crystalline symmetries. To illustrate this, let's first consider the case of a closed Fermi surface where only the $n = \pm 1$ harmonics are kept in the Fourier expansion. (One example where this holds exactly is an elliptical Fermi surface governed by the dispersion relation $\epsilon_{\mathbf{k}} = \frac{1}{2m}(k_x^2 + \alpha k_y^2)$ where $\alpha > 0$ reflects the degree of ellipticity).

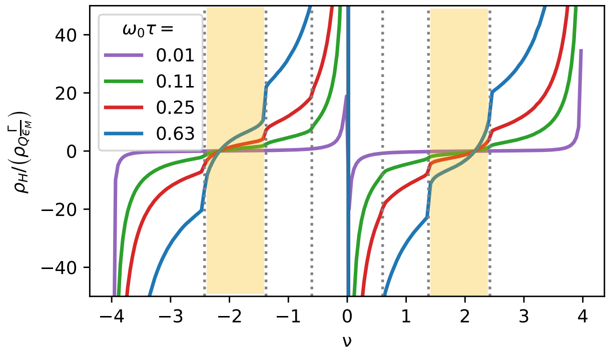

FIG. 5. Hall resistivity ρ_H at varying magnetic fields. Note that it crosses zero within the filling range of open Fermi surfaces on both sides of the charge neutrality point. These mark the sign-changing singularities in the Hall number depicted in Fig. 2(b) of the main text.

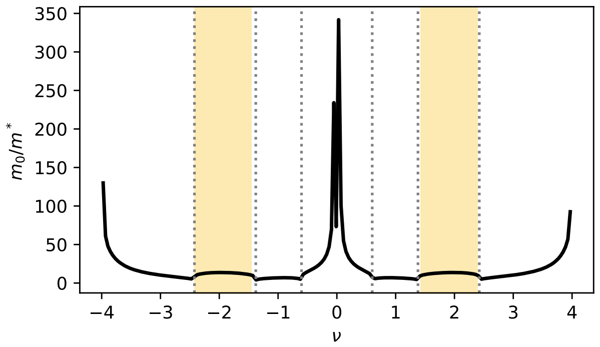

FIG. 6. Filling dependence of the averaged inverse cyclotron mass $1/m^*$, extracted from the averaged cyclotron frequency $\bar{\omega_c} = \frac{eB}{m^*}$. Here $\bar{\omega}_c = \frac{1}{N}\sum_{n,i}\omega_{c,n,i}$, where n and i label the FS (i) coming from a given band (n), in units of the bare electron mass. $1/m^*$ is larger near the charge neutrality and band edges, explaining the earlier onset of quantum oscillations in these filling regions.

Then the conductivity tensor in Eq. (21) of the main text reduces to:

$$\sigma = \frac{q^3 B}{\pi}\frac{\tau}{\omega_c}\begin{pmatrix} \frac{|v_1^x|^2}{1+\omega_c^2\tau^2} & \frac{\mathrm{Re}(v_1^x v_{-1}^y)-\mathrm{Im}(v_1^x v_{-1}^y)\omega_c\tau}{1+\omega_c^2\tau^2} \\ \frac{\mathrm{Re}(v_1^x v_{-1}^y)+\mathrm{Im}(v_1^x v_{-1}^y)\omega_c\tau}{1+\omega_c^2\tau^2} & \frac{|v_1^y|^2}{1+\omega_c^2\tau^2} \end{pmatrix}. \tag{5}$$

The off-diagonal piece contains both symmetric and antisymmetric components. The Hall resistivity ρ_H is given by the antisymmetric component of the resistivity tensor (obtained by inverting σ):

$$\rho_H = \frac{\pi}{q^3 B}\frac{\tau}{\omega_c}\frac{\omega_c\tau}{\mathrm{Im}(v_1^x v_{-1}^y)} \propto B. \tag{6}$$

Thus, if only the lowest harmonics are kept, the Hall resistivity is linearly proportional to the magnetic field, and the Hall number is therefore field-independent.

Crystalline symmetries lead to higher harmonics $n = \pm 2, \pm 3, \ldots$ in the Fourier expansion of the Fermi velocities $\mathbf{v}(s)$, and generally lead to B dependence of n_H. The B-dependence becomes more pronounced closer to the van Hove singularities, where the impact of crystalline symmetries on the band dispersion is more pronounced. Only in the high magnetic field limit ($\omega_c\tau \gg 1$) does the Hall number recover the constant-in-B dependence. This is again seen from Eq. (21) of the main text, which to leading order in $O(1/\omega_c\tau)$ is given by:

$$\sigma \approx \frac{q^3 B}{\pi}\frac{\tau}{\omega_c}\sum_{n=-\infty}^{\infty}\frac{1}{in\omega_c\tau}\begin{pmatrix} |v_n^x|^2 & v_n^x v_{-n}^y \\ v_n^y v_{-n}^x & |v_n^y|^2 \end{pmatrix} = \frac{q^3 B}{\pi}\frac{\tau}{\omega_c}\begin{pmatrix} 0 & \sum_{n=1}^{\infty}\frac{\mathrm{Im}(v_n^x v_{-n}^y)}{n\omega_c\tau} \\ -\sum_{n=1}^{\infty}\frac{\mathrm{Im}(v_n^x v_{-n}^y)}{n\omega_c\tau} & 0 \end{pmatrix}. \tag{7}$$

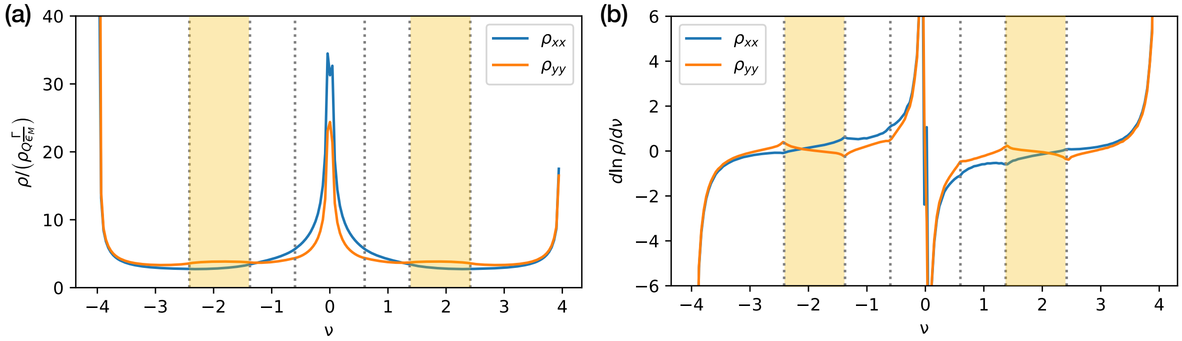

FIG. 7. (a) Longitudinal resistivitities ρ_{xx} (blue) and ρ_{yy} (orange) at $B=0$ for strained BM model, with $\epsilon = 0.2\%$ and $\varphi = 0°$. (b) The derivative of log resistivity with respect to filling. Gray dotted vertical lines mark positions of the van Hove points, and the yellow shaded area marks the open Fermi surface region.

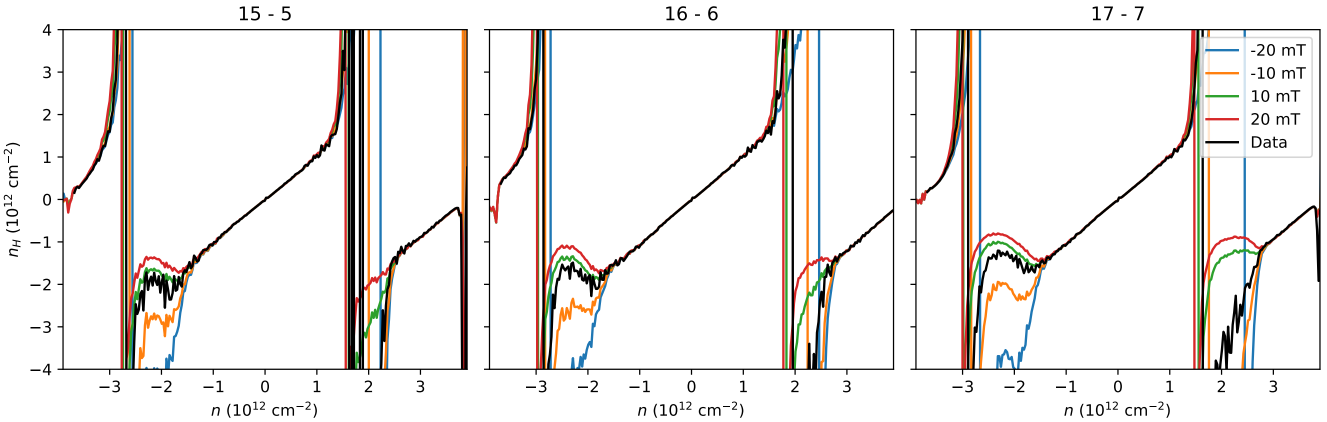

FIG. 8. Filling dependent Hall number corrected for antisymmetrization error of the indicated contact pairs at $B = 0.5$T. All other transverse contact pairs do not have a large degree of mixing and also do not have these features.

The matrix has the B-dependent matrix structure: $\sigma \propto \frac{1}{B}\begin{pmatrix} 0 & -1 \\ 1 & 0 \end{pmatrix}$. Inverting it gives $\rho_H(B) \propto B$ in the high field limit, and therefore a B-independent Hall number.

V. ERROR ANALYSIS OF ANTISYMMETRIZATION

In Fig. 8 we show that the bump-like features in the experimental Hall number plots in filling range of open Fermi surfaces (Fig. 2(d) of main text and Fig. 10 in the SM) may be attributed to improper antisymmetrization with respect to the B-field, namely,

$$\tilde{\rho}_H(B) = \frac{\rho_{yx}(B+\delta B) - \rho_{yx}(-B+\delta B)}{2}, \tag{8}$$

where δB is a systematic error. The error may be attributed to a small trapped flux of 10 mT in the superconducting magnet, or perhaps an offset in the magnet power supply. Due to the misalignment of transport principal axis with the Hall bar geometry, longitudinal MR also contributes to $\rho_{yx}(B)$. In the filling range with open Fermi surfaces, the longitudinal resistance exhibits non-saturating quadratic MR, and will mix into the Hall component which is odd in B. As a result, one expects the improper antisymmetrization error to be largest in this filling range.

We investigate this possibility by first fitting a polynomial to the low-field transverse resistivity. This allows us to interpolate the data and add small constant offsets prior to antisymmetrization. Accounting for an offset of roughly 20 mT largely removes the bumps from the data. This offset is larger than what we would expect from trapped flux

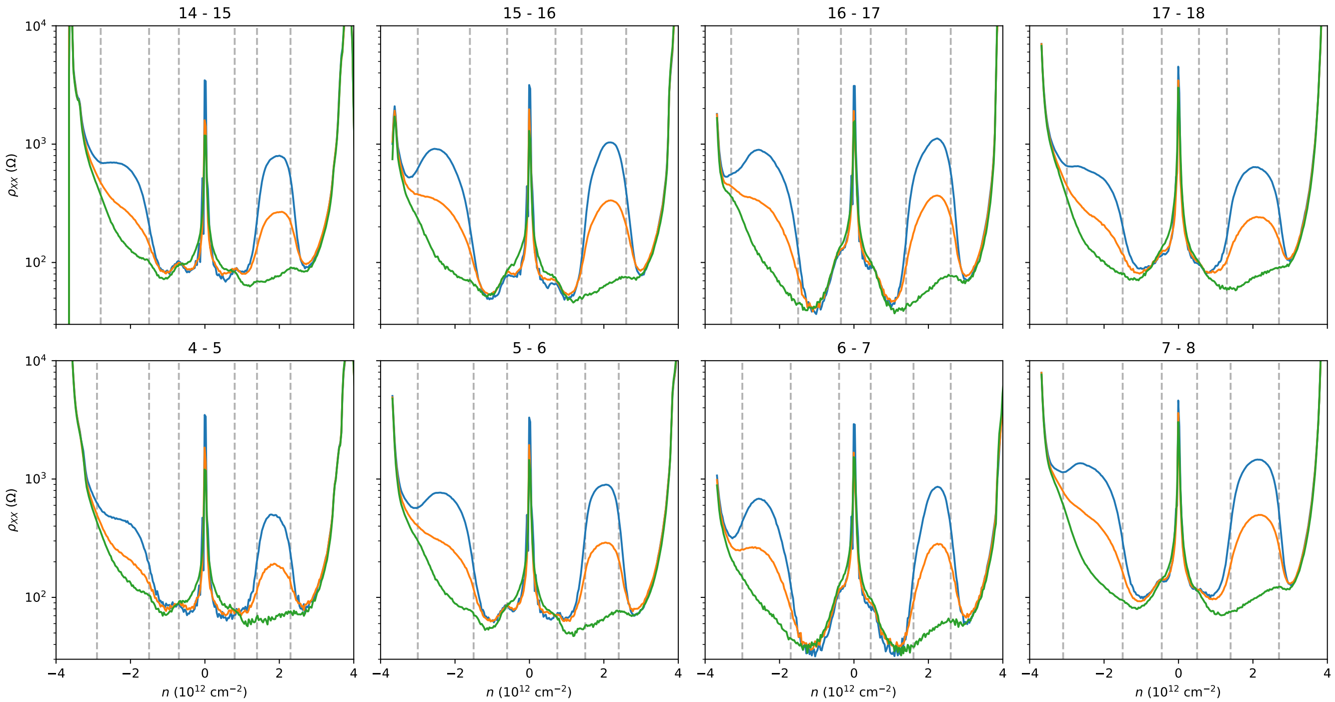

FIG. 9. All longitudinal contact pairs with quadratic MR at $B = 0$ (green), 0.25 T (orange), and 0.5 T (blue), symmetrized. Data taken at 1.6 K. Every contact pair has a well-developed shoulder or cusp near $n/n_s \approx \pm 0.5$ that we associate with the lowest-energy van Hove point. The additional vertical lines are by-eye guesses for the location of the other van Hove points.

in a superconducting magnet, however we do not expect the procedure to be accurate to such a fine degree, simply because we do not have fine enough resolution in field to get an accurate polynomial fit.

VI. MORE EXPERIMENTAL MEASUREMENTS BASED ON VARIOUS CONTACT PAIRS OF THE HALL BAR GEOMETRY

The device has nine voltage probes on each side. We observe quadratic magnetoresistance regions in roughly half of the device, between the fourth and eighth contacts. We present longitudinal resistivities of these pairs in Fig. 9. In each of these pairs, we observe behavior qualitatively consistent with that presented in the main text. Our Hall measurements (Fig. 10) are similarly consistent.

In Fig. 11, we show quantum oscillations and their Fourier transforms for all three contact pairs for which we have dilution refridgerator data. In all cases, we observe behavior consistent with what we present in the main text: 1) quantum oscillation onset at lower field close to CNP, 2) an irregular pattern of resistivity minima close to CNP, and 3) extra features in the FFT of the quantum oscillations that end at vH1. The density of the first van Hove point is closer to the CNP in the other two contact pairs, and the extra features in the FFT are not as clear.

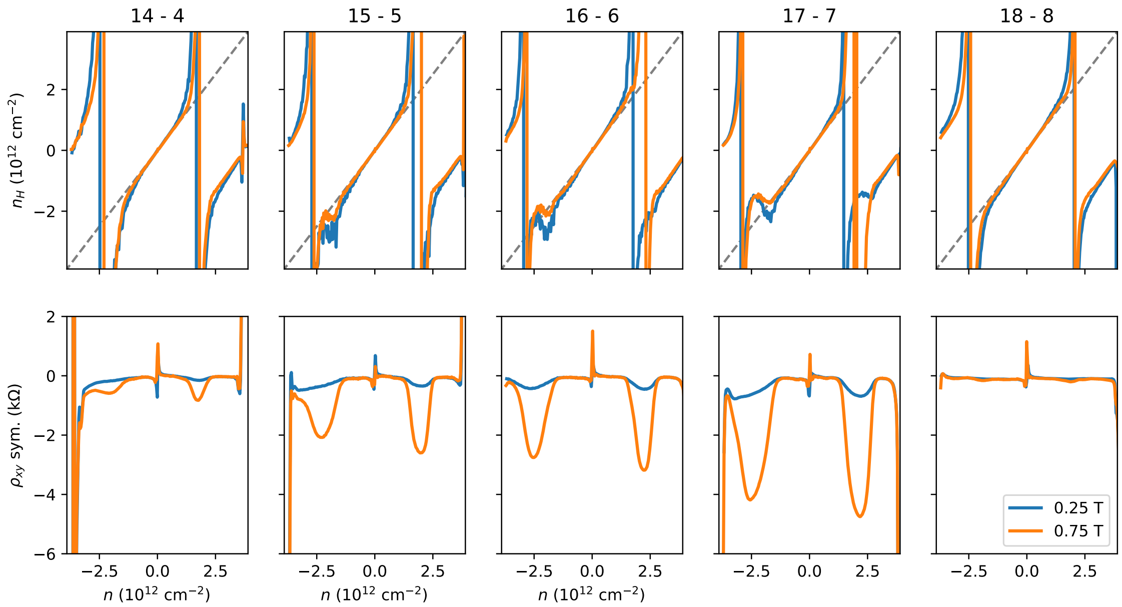

FIG. 10. Transverse contact pairs adjacent to the longitudinal pairs presented in Fig. 9, taken at 1.6 K. Top row: Hall number. Bottom row: symmetrized resistivity. The contact pairs with the largest symmetric component of magnetoresistance display bump-like features in Hall number near where they change sign, consistent with errors in antisymmetrization as illustrated in Fig. 8.

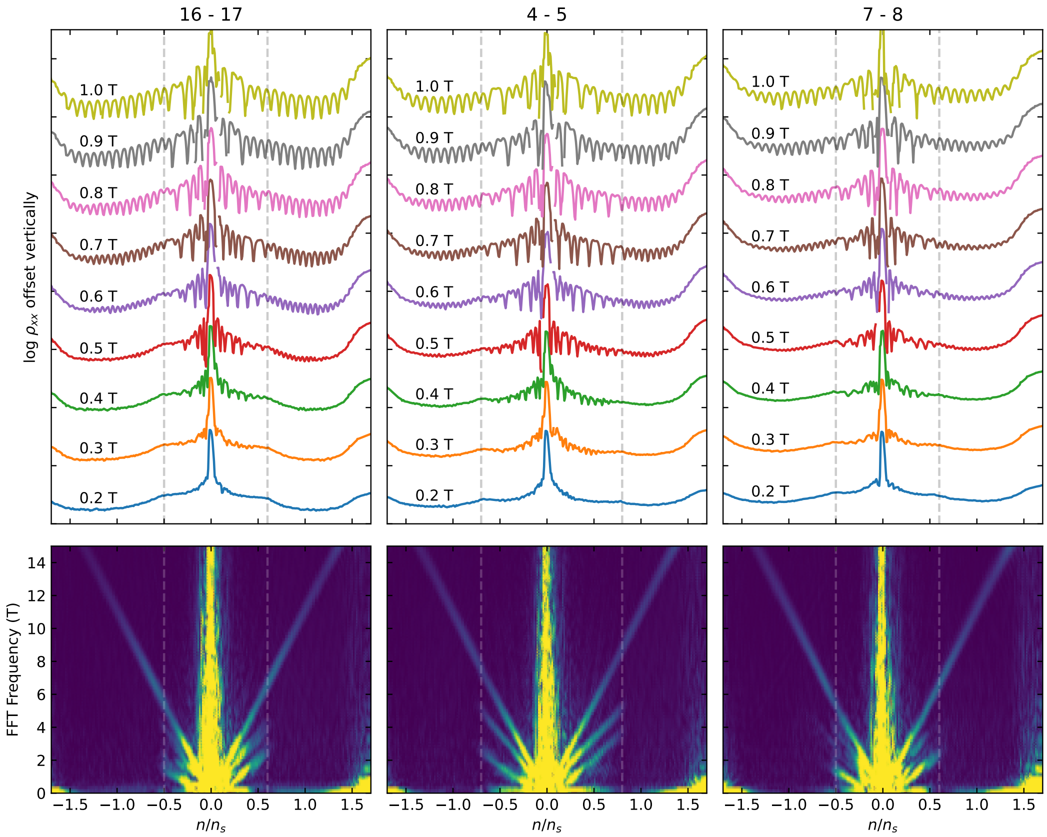

FIG. 11. Quantum oscillations near CNP for every longitudinal contact pair for which we have dilution refridgerator data (T = 26 mK). Vertical dashed lines are our estimate of the low-energy van Hove point based on cusps in resistivity at 0.2 T. Contact pair 4 - 5 is the pair shown in the main text.

[1] J. Finney, A. L. Sharpe, E. J. Fox, C. L. Hsueh, D. E. Parker, M. Yankowitz, S. Chen, K. Watanabe, T. Taniguchi, C. R. Dean, A. Vishwanath, M. A. Kastner, and D. Goldhaber-Gordon, Unusual magnetotransport in twisted bilayer graphene, Proceedings of the National Academy of Sciences **119**, e2118482119 (2022).

[2] A. Kerelsky, L. J. McGilly, D. M. Kennes, L. Xian, M. Yankowitz, S. Chen, K. Watanabe, T. Taniguchi, J. Hone, C. Dean, A. Rubio, and A. N. Pasupathy, Maximized electron interactions at the magic angle in twisted bilayer graphene, Nature **572**, 95 (2019).

[3] Z. Bi, N. F. Q. Yuan, and L. Fu, Designing flat bands by strain, Phys. Rev. B **100**, 035448 (2019).
